# Effect of childhood developmental coordination disorder on adulthood physical activity; Arvo Ylppö longitudinal study

**DOI:** 10.1111/sms.14144

**Published:** 2022-02-24

**Authors:** Jocelyn L. K. Tan, Anna‐Mari Ylä‐Kojola, Johan G. Eriksson, Minna K. Salonen, Niko Wasenius, Nicolas H. Hart, Paola Chivers, Timo Rantalainen, Aulikki Lano, Harri Piitulainen

**Affiliations:** ^1^ School of Health Sciences University of Notre Dame Australia Fremantle Western Australia Australia; ^2^ Western Australian Bone Research Collaboration Perth Western Australia Australia; ^3^ Department of Child Neurology Children’s Hospital University of Helsinki Helsinki Finland; ^4^ Folkhälsan Research Center Helsinki Finland; ^5^ Department of General Practice and Primary Health Care University of Helsinki Helsinki Finland; ^6^ Department of Obstetrics and Gynecology and Human Potential Translational Research Programme Yong Loo Lin School of Medicine National University of Singapore Singapore Singapore; ^7^ Singapore Institute for Clinical Sciences A*Star Singapore Singapore; ^8^ Unit of Chronic Disease Prevention Department of Public Health Solutions National Institute for Health and Welfare Helsinki Finland; ^9^ Institute for Health Research University of Notre Dame Australia Fremantle Western Australia Australia; ^10^ School of Medical and Health Science Edith Cowan University Joondalup Western Australia Australia; ^11^ Centre for Healthcare Transformation Queensland University of Technology Brisbane Queensland Australia; ^12^ Gerontology Research Center University of Jyväskylä Jyväskylä Finland; ^13^ Faculty of Sport and Health Sciences University of Jyväskylä Jyväskylä Finland; ^14^ Department of Neuroscience and Biomedical Engineering School of Science Aalto University Aalto Finland

**Keywords:** accelerometry, developmental disability, motor competence

## Abstract

Individuals at risk of Developmental Coordination Disorder (DCD) have low levels of physical activity in childhood due to impaired motor competence; however, physical activity levels in adulthood have not been established. This study sought to determine the impact of DCD risk on physical activity levels in adults using accelerometry measurement. Participants (*n* = 656) from the Arvo Ylppö Longitudinal Study cohort had their motor competence assessed at the age of five years, and their physical activity quantified via device assessment at the age of 25 years. Between group differences were assessed to differentiate physical activity measures for individuals based on DCD risk status, with general linear modeling performed to control for the effects of sex, body mass index (BMI), and maternal education. Participants at risk of DCD were found to have a lower total number of steps (*d* = 0.3, *p* = 0.022) than those not at risk. Statistical modeling indicated that DCD risk status increased time spent in sedentary light activity (β = 0.1, 95% CI 0.02 to 0.3, *p* = 0.026) and decreased time spent in vigorous physical activity via interaction with BMI (β = 0.04, 95% CI 0.001 to 0.1, *p* = 0.025). Sensitivity analysis found that visuomotor impairment did not significantly impact physical activity but did increase the role of DCD risk status in some models. This 20‐year‐longitudinal study indicated that DCD risk status continues to negatively impact on levels of physical activity into early adulthood.

## 1 INTRODUCTION

Individuals with motor difficulties, manifesting clinically as developmental coordination disorder (DCD) in approximately five percent of the population, have difficulties with the performance of their motor skills to a degree that is impactful upon everyday functioning.^1^ In 75 to 80% of cases with DCD motor difficulties recognized in childhood persist into adulthood^1^ and although natural variation of motor competence in early childhood prevents diagnosis of DCD prior to the age of five, the presence of motor difficulties indicating DCD risk in preschool‐aged children has been shown to be a good indicator of persistent motor difficulties.^2^

Preschool‐aged children at risk of DCD have been identified to have physical activity deficits,^3^ similar to those reported throughout childhood and adolescence for individuals with DCD.^1^, ^4^, ^5^ As motor deficits associated with DCD usually continue into adulthood, along with negative physical activity beliefs^4^ and the use of avoidance‐based coping mechanisms^6^ continued detriment of physical activity into adulthood would be anticipated. Although this has been reported via self‐report,^6^ there is currently an absence of device‐assessed measures of physical activity in this group. This absence is particularly pertinent, as studies in pediatric populations have reported a discrepancy between self‐report and device‐assessed measures of physical activity in children with DCD^7^ and as such physical activity self‐reports in adults need confirmation. Due to the prevalence of DCD, continued low physical activity could have population level health repercussions given the increased risk of sedentary behavior‐related chronic conditions later in life,^8^, ^9^ and markers for these conditions have been reported in adults with DCD.^10^ As such, the absence of device‐assessed measures of physical activity in an adult population with DCD is a significant gap in the literature with the potential for significant health implications.

In quantifying the differences in physical activity in adults with childhood DCD risk, the role of specific areas of impairment as a barrier to physical activity is a necessary avenue for investigation. Studies of physical activity in pediatric populations report varying levels of deficit,^7^ which may in part be due to the impact of a variety of factors known to impact upon physical activity such as gender, body mass index (BMI), and socioeconomic factors.^11^ However, a specific area affecting physical activity for individuals at risk of DCD is the frequent co‐occurrence of impairments outside of pure motor competence issues,^1^, ^12^ which may also act to impair physical activity. A common deficit among individuals at risk of DCD is visuomotor integration (VMI),^13^ the coordination of visual and motor‐related neuronal processing known to impact behavior and perception.^13^ Individuals with DCD and VMI deficits have been shown to have different areas of motor deficit than those with motor competence impairment only^14^, ^15^ and decreasing diversity and intensity of physical activity with increasing VMI deficits has been shown in children with DCD.^16^ It is not known whether VMI plays a similar role for adults with a history of DCD risk; however, prior work using the Arvo Ylppö Longitudinal Study (AYLS) population established a link between decreased VMI and negative health outcomes in the form of increased body fat percentage and increased body mass index (BMI)^17^ of which lower levels of physical activity could be a causative factor. The potential for VMI impairments to reduce physical activity indicates a need for further investigation of the role of VMI on physical activity in a DCD population.

This study aims to describe the relationship between childhood DCD risk status and VMI deficits defined at the age of approximately 5 years, and physical activity levels recorded at the age of 25 years in a young adult population by addressing the following two questions:

(1) Does early DCD risk status have an impact upon physical activity levels into early adulthood?
(2) Does early VMI impairment have an impact upon physical activity levels into early adulthood, either independently or in combination with DCD?

It was hypothesized that both DCD risk status and VMI impairment will have a negative long‐term effect on the physical activity levels (increased sedentary behavior, decreased moderate to vigorous physical activity compared to nonaffected referents) that would still be evident at the age of 25 years.

## 2 METHODS

### 2.1 Experimental design

This is an analysis of participants from the AYLS, a longitudinal prospective cohort study.^18^ The current study explores the impact of DCD status and VMI impairment at the age of approximately five years on physical activity at the age of 25 years using data from birth, 56 months, and 25 years. DCD risk status via motor competence assessment and VMI using the Beery scale were assessed at the age of 56 months. Participants had anthropometry assessment (height and weight), and accelerometry performed at the age of 25 years.

### 2.2 Participants

**FIGURE 1:**
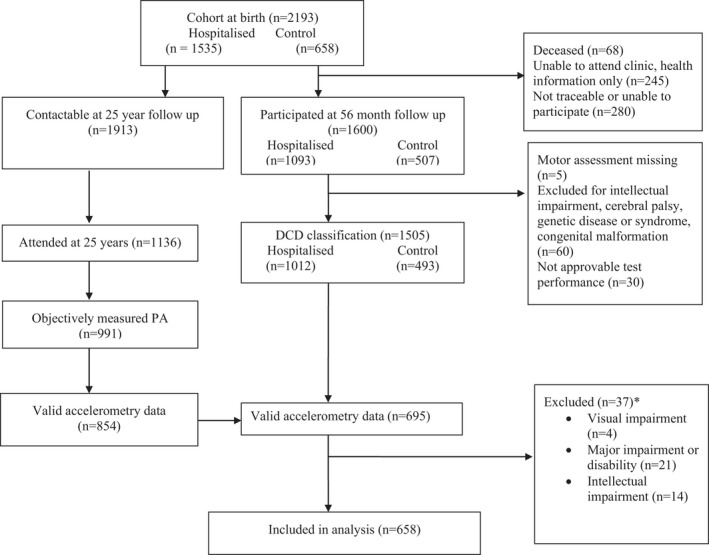
Participant flow through study, including exclusion points. *Some participants qualified for exclusion on more than one criterion

The AYLS comprised of infants born alive from seven maternity hospitals in the county of Uusimaa, Finland between March 15, 1985, and March 14, 1986. A total of 1535 participants were recruited who had been admitted to neonatal wards of obstetric units or the Neonatal Intensive Care Unit of Children's Hospital, Helsinki University Hospital, Finland, within ten days of their birth, with an additional 658 healthy control infants prospectively and randomly recruited via three maternity hospitals. Participants were invited to clinical follow‐up visits at age 56 months and 25 years. As shown in Figure 1, some participants attended at one clinical follow‐up visit only, with about twenty percent of those with valid accelerometery data not attending at the age of 56 months which is considered to be due to the mobility of the sample. Missing data analysis of participants who had valid accelerometery data at the age of 25 years found no significant differences in gender, hospitalization rate, parental education level, birthweight, gestational age, or in sum scores for obstetric or neonatal optimality when assessed based upon attendance at 56 months. However, participants who were included in DCD classification at 56 months but did not have accelerometery performed at the age of 25 years were found to be more frequently male (57.5% compared to 48.9%, χ^2^ = 11.2, *p* < 0.001), hospitalized following birth (70.5% compared to 63.5%, χ^2^ = 8.4, *p* = 0.004), and had parents with a lower education level (maternal χ^2^ = 20.2 *p* < 0.001; paternal χ^2^ = 12.7 *p* = 0.005). The childhood protocol was approved by the ethics committees of the Women's Hospital and Children's Hospital of Helsinki University Hospital, the Helsinki City Maternity Hospital, and Jorvi Hospital, and in adulthood by the Coordinating Ethics Committee of the Helsinki and Uusimaa Hospital District. Informed consent was provided by parents in childhood and participants in adulthood.

The current study reports on a subsample of 695 participants drawn from the AYLS cohort. Participants were excluded from analysis if they had an impairment that could impact upon their motor skills in accordance with criterion D of the DSM‐V criteria for DCD diagnosis^19^ as reported by parents or their medical records. Reasons for exclusion included intellectual impairment, cerebral palsy, genetic disease, and congenital malformations (Figure 1). An additional four cases were excluded as they had visual impairment to a degree that may have impacted upon their VMI score.

### 2.3 Assessment measures and tools

#### 2.3.1 Motor competence testing

Motor competence was assessed by four experienced pediatricians (incl.AL) of the research team using a quantitative test of motor competence developed for the AYLS study. The test contained items similar to the Zurich Neuromotor Assessment,^20^ and each child was scored on whether their performance on each item was within normal range. Individual test items are listed in Appendix A. The Zurich Neuromotor assessment is designed for use in children from the age of five years, although adjusted versions of the test have been found to be reliable in children aged three to five.^21^ Test–retest correlations are between 0.66 to 0.80 in children aged between five and ten years of age^21^ and convergent validity with other motor tests established.^21^

As some children refused to perform all tasks, a percentage sum score of successful tasks to attempted tasks ($\frac{n\left(succesful\;tasks\right)}{n(attempted\;tasks)}\times 100$)^22^ was used to define the child's motor competence.^23^ For children who made insufficient task attempts (less than seven), the calculated percentage score on attempted tasks was only used if the score was outside of normal range, children who had insufficient attempts but whose percentage score was within normal range were excluded from analysis.^23^ DCD risk status was established based on the cutoff points where five percent and fifteen percent of the healthy control subjects in the original AYLS study population (*n* = 493) failed, equivalent to a score of 68.75 and 78.95, respectively. Due to the children being below diagnostic age for DCD at the time of testing, the groups were classified as “at risk of DCD” (DCD5) at the five percent cutoff and “probably at risk of DCD” (DCD15) at the fifteen percent cutoff.^24^ The impact of motor skills upon activities of daily living was assessed via parental clinical interviews at child age 4.7 years, including questions on age‐appropriate activities of daily living (e.g., buttoning, dressing self), social relationships, play skills, and motor skill performance (running, catching a ball, riding a bike).

#### 2.3.2 Visuomotor integration (VMI) testing

VMI was assessed using 12‐items of Beery‐Buktenica Developmental Test of Visual Motor Integration where children are instructed to copy geometric forms which increase in complexity.^25^ Test scores were corrected for exact age at measurement and converted to have a mean of 100 and a standard deviation of 15, such that standardized scores represent the difference from the mean for healthy children born at term. The Beery VMI has convergent validity with other tests of visual perception^26^ and a reported inter‐rater reliability of 0.92, internal consistency of 0.96, and test–retest reliability of 0.89.^27^ For consistency with DCD categorizations, VMI scores were categorized into the bottom 5th percentile, and 5–15th percentile of scores, corresponding to cutoff scores of 75.6 and 82.6, respectively.

#### 2.3.3 Quantification of physical activity

Physical activity was measured with SenseWear Pro 3 Armband (Body Media, Inc., Pittsburgh, PA, USA), a multisensory body monitor including a two‐way axis accelerometer.^28^ The SenseWear Armband has been found to be valid for physical activity measurements in young adults in resting conditions, exercise conditions, and field monitoring.^29^, ^30^ Participants were instructed to wear the armband on their right triceps for ten consecutive days. Participants were included if they had more than three valid days, weekday or weekend, with a valid day having more than ten hours of wear. This criterion was designed to maximize sample size while providing measurement reliability.^31^, ^32^ The device logged physical activity based on the acceleration recordings minute by minute, which was combined with subject's characteristics such as gender, age, and BMI to estimate intensity of physical activity, distance of data points from the mean (mean amplitude deviation), and number of steps, using manufacturer algorithms (SenseWear Professional Software, v6.1). Following the removal of any measurements indicated by the device to be sleep, each minute was classified into sedentary light (under 3 metabolic equivalent[MET]), moderate (3 to under 6 MET), vigorous (6 to under 9 MET), or very vigorous (above 9 MET).^28^ Vigorous and very vigorous minutes were pooled into the vigorous category, and a moderate‐vigorous category (MVPA) created by pooling moderate and vigorous categories. Mean durations in minutes per day are reported as the outcome. Physical activity was assessed as minutes per day, and percentage of total wear time. Minutes per day for MVPA was converted to a weekly duration by multiplying by seven, which was then categorized to determine whether participants met World Health Organisation (WHO) Guidelines for physical activity. Cutoffs for meeting guidelines were set at 150 minutes for MVPA, covering minimum requirements for moderate and vigorous activity.^33^

#### 2.3.4 Anthropometric and background measures

Researchers collected information about pre‐, peri‐, and neonatal conditions from medical records on daily ward visits. Information about parental educational status was collected via parental interviews at wards and 56‐month clinical visits. Anthropometric measures for height in centimeters and weight in kilograms were taken by trained research nurses during clinical visits at 56 months and 25 years. Height was measured to the nearest 0.1cm and weight in light indoor clothing to the nearest 0.1kg. As some participants did not attend at the exact age for each visit, corrections were made for exact age by linear regression. BMI was calculated as weight (kg)/height (m)^2^ and categorized into weight status for age and gender using the WHO standards for childhood measures^34^ and the Centre for Disease Control standards for adult measurements.^35^

#### 2.3.5 Data analysis

All analysis was performed in IBM SPSS, version 26, excepting effect size measures which used the Psychometrica online calculator.^36^ Alpha was set at 0.05. All variables were assessed for normality using visual assessment and Shapiro–Wilk test. Data were assessed to be missing at random. Descriptive between group differences for confounders by risk group were assessed using either an independent t‐test, Kruskal–Wallis, Mann–Whitney U, or chi‐square tests. Between group differences were assessed for age, BMI, and accelerometery via Mann–Whitney U as the data had a non‐parametric distribution. BMI categories, change in BMI categories between time points, and meeting of physical activity guidelines were assessed via chi‐square analysis. Age, BMI, and accelerometery measurements were described using mean (M), median (Md), and standard deviation (SD). Parental age, birthweight, gestational age, and VMI scores were described with M and SD. Pre‐, peri‐, and neonatal risk factors as well as socioeconomic factors as reflected by parental (paternal and maternal) education level were described as frequencies in each risk category. Motor competence measures were described as both group frequencies for anomalous measures and M, Md, and SD for continuous scores. Cohen's *d* effect sizes were calculated and classified as small *d* = 0.2, medium *d* = 0.5, and large *d* = 0.8. As following assessment, no significant difference was shown between the DCD5 and DCD15 categories, and in accordance with International Clinical Practice recommendations where the 16th percentile is set as a cutoff for DCD,^1^ the groups were combined into a single risk category (DCD) and general linear modeling was done at this level. Accelerometery and BMI measurements were performed for the entire risk group, as well as at the 5th and 15th percentile, while confounder assessment was done at the 5th and 15th percentile only.

The relationship of VMI and DCD category with physical activity levels was explored using a general linear model. Predictors included in the final model were sex, BMI, socioeconomics as reflected by mother's educational attainment, DCD or VMI category, and an interaction variable between risk category and BMI. Three other models were also conducted: Model one included predictors of sex and risk category only, model two included predictors of sex, BMI, and risk category, and model three contained predictors of sex, BMI, mother's educational attainment, and risk category. The interaction variable predictor was included after prior models indicated that the addition of BMI removed the effect of risk category. Figures of the interaction effect were derived from the final model presented in the manuscript. All other predictors were chosen as significant predictors for physical activity via accelerometery in young adults based on prior literature,^11^ with mother's educational attainment included as it is the most commonly used indicator of socioeconomic status.^37^ Age was not included in the model as the mean between group difference in age at time of accelerometery was 0.7 months and hence not clinically relevant at the age of 25 years. The final model was chosen based on Akaike information criterion (AIC), with the most complex model showing the best AIC fit. Residual plots for each model were visually assessed and determined to violate the assumption of normality, and as such, accelerometery data were transformed via natural log. Model residuals for the transformed data showed no violations although slight deviations were seen in the tails of some models. Due to reported sex effects on physical activity in this group,^5^ subgroup analysis was performed limiting the analysis by sex. A sensitivity analysis was also performed to determine the effects of using a minimum of three rather than four days as inclusion criteria in order to maximize sample size.

## 3 RESULTS

### 3.1 Motor competence

#### 3.1.1 Motor competence measures

Motor competence testing indicated 30 participants (23 male, 7 female) as DCD5, with an additional 53 participants (43 male, 10 female) being categorized as DCD15, and 575 participants (250 male, 325 female) as no‐risk. Both risk groups (DCD5 M = 88.1 [SD = 13.91, MD = 89.7]; DCD15 M = 97.0 [SD = 11.9, Md = 96.8]) showed detriments in their VMI score compared to the no‐risk group (M = 102.1 [SD = 14.1, Md = 103.9]). These differences were statistically significant when compared at the 5th percentile (t = −4.9, *p* < 0.001) and the 15th percentile (t = −4.8, *p* < 0.001) to the no‐risk group. DCD risk groups were shown to have increased difficulty with motor skill performance at 5 years old with a higher proportion of the at‐risk group being reported to have difficulties in ball catching (DCD5 36.7%; DCD15 30.2% compared to 14.1% in no‐risk, χ^2^ = 18.4, *p* < 0.001) and running (16.7% DCD5; 5.7% DCD15 vs. 2.6% in no‐risk, χ^2^ = 17.5, *p* < 0.001).

#### 3.1.2 Background variables

Motor competence groups were of similar health levels at birth with no differences detected in infant or maternal risk factors (Table 1), including gestational age. No differences between DCD groups were detected for parental education maternally (χ^2^ = 3.9, *p* = 0.685) or paternally (χ^2^ = 5.4, *p* = 0.496). No difference in adiposity as assessed by BMI was found between groups in either score or corresponding category at either five or 25 years of age, although the group as a whole increased in adiposity with a total of 32.7% being overweight or obese at age 25 compared to 15.8% at age five. Change in adiposity as indicated by BMI category change between five‐year assessment and 25‐year assessment did not detect a difference for the DCD5 group (χ^2^ = 1.1, *p* = 0.896) nor the DCD15 group (χ^2^ = 2.7, *p* = 0.604). Between group differences at 5 years of age are shown in Table 2.

**TABLE 1:** Prenatal, perinatal, and neonatal characteristics by DCD risk category

	DCD5	DCD15	Not at risk	Group difference	*p*
	%	%	%	χ^2^	
Pre and perinatal risk factors					
Maternal severe chronic illness	6.7	9.4	5.6	1.3	0.513
Multiple pregnancy	6.7	1.9	4.9	1.2	0.539
Pre‐eclampsia	23.3	11.3	12.0	3.4	0.180
Fetal distress during pregnancy	10.0	5.7	7.3	0.5	0.766
Fetal distress during birth	26.7	15.1	16.2	2.4	0.307
Small for gestational age	6.7	3.8	6.1	0.5	0.780
Neonatal risk factors/complications					
Hospitalized	56.7	69.8	62.3	1.7	0.437
Intubation or ventilator treatment	10.0	7.5	9.4	0.2	0.898
Suspicion/verified septic infection	6.7	5.7	5.7	0.05	0.977
Surgical operation	3.4	1.9	1.2	1.1	0.588
Severe anemia requiring blood transfusion	6.7	3.8	4.0	0.5	0.767
Apnea	6.7	3.8	2.3	2.5	0.283
Clinical seizures	0.0	0.0	1.6	1.3	0.518
IVH grade 1–2	3.3	0.0	1.1	2.0	0.364

**TABLE 2:** Characteristics at 56 months follow‐up

	DCD5	DCD15	Not at risk	Group difference	
	M (SD)	M (SD)	M (SD)	H	*p*
Age (y)	4.7 (0.05)	4.7 (0.03)	4.7 (0.04)	1.2	0.547
Weight (kg)	18.5 (3.3)	18.4 (2.5)	18.2 (2.5)	20.0	<0.001
BMI	15.7 (2.1)	15.5 (1.5)	15.4 (1.3)	0.6	0.748
VMI (% sum score)	88.1 (13.9)	97.0 (11.9)	102.1 (14.1)	32.2	<0.001

	%	%	%	χ^2^	*p*
Hardly able to catch a ball	36.7	30.2	14.1	18.4	<0.001
Running, only slowly	16.7	5.7	2.6	17.5	<0.001
BMI grouping					
Underweight	3.3	1.9	1.4	9.5	0.149
Healthy	73.3	76.9	83.9		
Overweight	13.3	19.2	12.4		
Obese	10.0	1.9	2.3		

### 3.2 Visuomotor integration (VMI) measures

#### 3.2.1 Visuomotor integration (VMI)

Division of groups based on VMI testing found 23 participants (16 male, 7 female) in the bottom 5th percentile, 32 (18 male, 14 female) in the 5th to 15th percentile and 579 above the 15th percentile (272 male, 309 female), with no difference in motor competence (<5th percentile M = 98.2[SD = 4.0], 5 to 15th percentile M = 99.0 [SD = 2.3], >15th percentile M = 99.2 [SD = 2.5], H = 5.0, *p* = 0.083).

#### 3.2.2 Background variables

The VMI groups showed some significant differences in risk factors in the neonatal period with those with lower scores having more neonatal complications and a lower gestational age. These differences are shown in Appendix B. VMI category did not impact on BMI or BMI category but impacted upon BMI change, with a significant difference being found for those in the bottom 15th percentile of VMI compared to those above the 15th percentile. The ≤15th percentile group was more likely to change category both down (18.8% ≤15th percentile vs 10.6% >15th percentile) and up (30.2% ≤15th vs. 26.5% >15th percentile) compared to those above the 15th percentile (χ^2^ = 15.0 *p* = 0.005).

### 3.3 Physical activity

At 25 years of age, between group difference tests for the entire DCD group showed fewer steps taken compared to the no‐risk group (Md = 9083.4 compared to Md = 9927.9, *d* = 0.3, U = 20161.0, *p* = 0.022). The entire DCD group spent a higher proportion of time in sedentary light physical activity than the no‐risk group constituting a mean of 62.8% of their total measured time (SD = 6.0, Md = 63.7) compared to 61.2% for the no‐risk group (SD = 6.4, Md = 61.8) (U = 20 205.0, *d* = −0.3, *p* = 0.024). This difference in sedentary physical activity was also found in the DCD15 group for proportion of time in sedentary light activity (Md = 63.7 compared to Md 61.8, *d* = −0.3, U = 20 205.0, *p* = 0.024) and total sedentary physical activity (M = 872.1 minutes [SD = 92.6, Md = 877.5] vs. M = 836.5 [SD = 105.3, Md = 853.7]) (*d* = −0.3, U = 12272.0, *p* = 0.019). No other differences in physical activity measures were detected between the DCD5 and no‐risk group. No differences were found between risk groups in the frequency of participants meeting WHO physical activity guidelines for MVPA.^33^ Subgroup analysis restricting by sex found that no physical activity differences were statistically significant based on DCD risk status for either sex when analyzed separately. Between group difference measures are reported in Tables 3 and 4. Of the eight participants with only three days measurement, one was in the DCD5 group and two in the DCD15 group; however, there was no significant difference between risk groups for number of days included or total number of minutes recorded. A sensitivity analysis removing participants with less than four recorded days found no significant effect on any analysis, aside from the model for DCD risk and sedentary light activity. Results from sensitivity analysis are detailed in Appendix G.

**TABLE 3:** Accelerometry differences between DCD risk groups

	DCD5 *N* = 30	DCD15 *N* = 53	Not at risk *N* = 573	H statistic	*p*
	M (SD)	M (SD)	M (SD)		
Sedentary Light (min/day)	843.2 (120.8)	872.1 (92.6)	836.5 (105.3)	5.4	0.067
Moderate (min/day)	129.6 (69.3)	130.7 (65.5)	139.1 (79.0)	0.4	0.802
Vigorous (min/day)	5.4 (6.3)	6.4 (7.8)	6.6 (8.2)	0.2	0.889
MVPA (min/day)	135.0 (71.1)	137.0 (68.3)	145.7 (82.5)	0.5	0.796
% Sedentary light activity	62.9 (6.2)	62.8 (5.9)	61.2 (6.4)	5.2	0.074
% Moderate activity	9.5 (4.8)	9.4 (4.6)	10.2 (5.7)	0.7	0.713
% Vigorous activity	0.4 (0.5)	0.5 (0.6)	0.5 (0.6)	0.2	0.918
% MVPA	9.9 (5.0)	9.8 (4.8)	10.7 (6.0)	0.7	0.714
Steps	9136.1 (3205.3)	9436.9 (3430.3)	10335.8 (3642.4)	5.3	0.070
Mean amplitude deviation	0.96 (0.3)	0.96 (0.3)	0.99 (0.3)	0.9	0.633

**TABLE 4:** Accelerometry group difference between DCD (DCD5 and 15) and not at risk

	DCD *N* = 83	Not at risk *N* = 573	Group difference		
	M (SD)	M (SD)	*d*_Cohen_	U‐statistic	*p*
Age (y)	24.9 (0.6)	24.8 (0.7)	−0.1	−1.1^a^	0.267
BMI	25.1 (5.1)	23.9 (4.2)	−0.03	20342.0	0.030
Sedentary light (min/day)	861.7 (103.9)	836.5 (105.3)	−0.2	20831.0	0.061
Moderate (min/day)	130.3 (66.5)	139.1 (79.0)	0.1	22827.0	0.522
Vigorous (min/day)	6.0 (7.3)	6.6 (8.2)	0.07	23522.0	0.833
MVPA (min/day)	136.3 (68.9)	145.7 (82.5)	0.1	22796.5	0.510
% Sedentary light activity	62.8 (6.0)	61.2 (6.4)	−0.3	20205.0	0.024
% Moderate activity	9.4 (4.7)	10.2 (5.7)	0.1	22646.0	0.452
% Vigorous activity	0.4 (0.5)	0.5 (0.6)	0.2	23444.0	0.796
% MVPA	9.9 (4.8)	10.7 (6.0)	0.05	22613.0	0.440
Steps	9328.2 (334.2)	10335.8 (3642.4)	0.3	20161.0	0.022
Mean amplitude deviation	0.96 (0.3)	0.99 (0.3)	−0.03	22352.0	0.351

^a^ *t*‐test.

**FIGURE 2:**
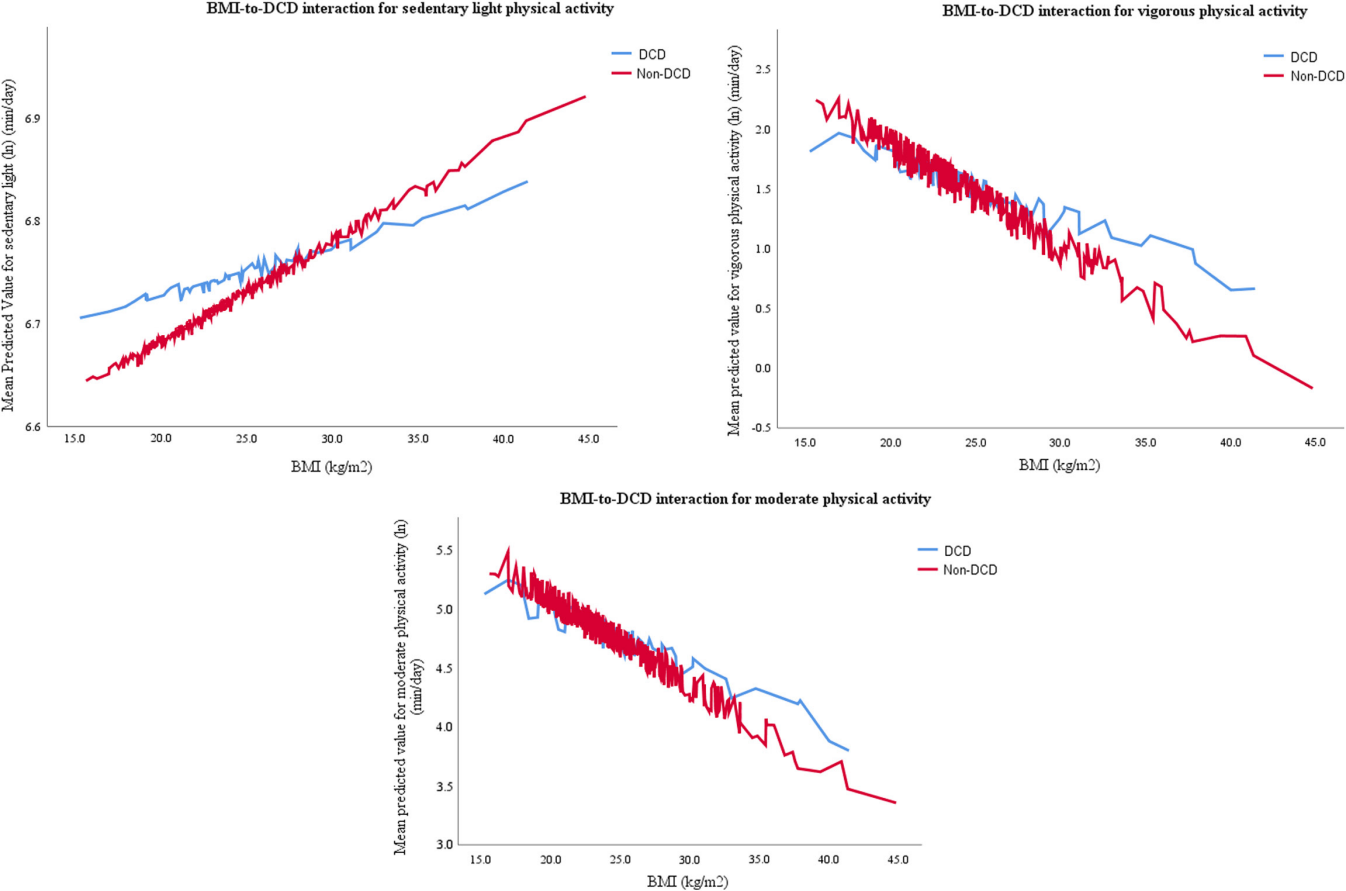
Interaction effect between DCD risk status and BMI for GLM models for physical activity, both with and without VMI as a continuous variable, showing a slower rate of change in physical activity for participants classed as DCD compared to those who were not. Sedentary light and vigorous models include VMI, moderate model does not

GLM modeling of physical activity variables showed a significant role for the DCD group in sedentary light physical activity (β = 0.1, *p* = 0.027) when sex, BMI, DCD risk, maternal education, and BMI‐to‐DCD interaction were included in the model, as shown in Appendix C. A statistically significant role was also seen in the sedentary light model for BMI (β = 0.01, *p* < 0.001) and a non‐significant effect for BMI‐to‐DCD interaction (β = −0.01, *p* = 0.057). The BMI‐to‐DCD effect became significant in sensitivity analysis when participants with less than four recorded days were removed (β = −0.01, *p* = 0.048). The interaction, depicted in Figure 2, was such that the non‐DCD group increased time spent in sedentary light activity at a faster trajectory than the DCD group. The model for vigorous physical activity suggested a role for DCD via its interaction with BMI (β = 0.04, *p* = 0.050), although not significant, with an additional non‐significant role for DCD risk category (β = −0.9, *p* = 0.062). This model, shown in Figure 2, showed time spent in vigorous physical activity decreased at differing rates between groups with the non‐DCD group losing more time in vigorous physical activity as BMI increased than the DCD group.

Models including VMI as a continuous variable, shown in Appendix D, found a statistically significant effect for DCD risk in more models, although no significant effect was detected for VMI. DCD had a statistically significant effect in the models for sedentary light activity (β = 0.2, *p* = 0.007), moderate activity (β = −0.6, *p* = 0.020), and MVPA (β = −0.7, *p* = 0.014) such that sedentary light activity levels were higher for those in the DCD group while moderate and MVPA levels were lower. A DCD‐to‐BMI interaction was seen in models for sedentary light activity (β = −0.01, *p* = 0.019), moderate activity (β = 0.03, *p* = 0.027), and MVPA (β = 0.03, *p* = 0.018), illustrated in Figure 2, which resulted in a more rapid reduction/increase in physical activity with increasing BMI.

Sensitivity analysis to determine whether VMI risk had similar impact to DCD status on accelerometry found that the <5th percentile group indicated less vigorous activity (Md = 1.1 compared to Md = 3.8 minutes a day) when compared to the >15th percentile group (*d* = 0.2, U = 4780.0, *p* = 0.021), while the 5 to 15th percentile showed reduced moderate physical activity compared to the >15th percentile group (Md = 156.9 minutes a day compared to Md = 121.2, *d* = 0.3, U = 7349.0, *p* = 0.046). Combining the groups to <15th percentile found no significant differences on any physical activity measure. No significant differences were found between risk groups in frequency of meeting physical activity guidelines. Subgroup analysis by sex found differing physical activity effects for each sex, with the vigorous activity effect for the <5th percentile group being only significant for males, and the 5 to 15th percentile reduced moderate physical activity differences only being significant for females. Additional significant effects were found for females only at the 5 to 15th percentile of reduced MVPA, percentage time in moderate and MVPA, and total steps (Appendix F). GLM modeling of <15th percentile did not detect a significant role for VMI risk in any model, although VMI‐to‐BMI interaction effect was significant in the model for mean amplitude deviation, such that mean amplitude deviation decreased more rapidly with increasing BMI for the VMI risk group. Between group difference test and model results for VMI scores can be found in Appendix [Link], [Link].

## 4 DISCUSSION

DCD risk status in early childhood was found to impact upon some aspects of physical activity in early adulthood, with a small to medium effect on total steps and sedentary light physical activity. Controlling for VMI impairment further increased the role of DCD risk in statistical models but an independent role for VMI was not shown. As such, DCD risk status in childhood appears to have a role in impairing some aspects of physical activity and individuals which may be influenced by the co‐occurrence of detriments in VMI.

### 4.1 Does early DCD risk status impact upon physical activity levels at the age of 25?

This study found DCD risk status in childhood impacted upon some aspects of physical activity in early adulthood. Between group differences were evident for the entire DCD risk group who took fewer total steps and spent a higher percentage of their day in sedentary light activity compared to their non‐DCD counterparts. Statistical modeling controlling for the effects of sex, BMI, and maternal education also found an increase in the number of minutes per day in sedentary light physical activity for the DCD group. These findings extend what has been found in pediatric DCD accelerometery studies^4^, ^7^, ^38^ providing device measured evidence to confirm that the physical activity pattern shown in individuals at risk for DCD during childhood extends into at least early adulthood. Deficits in motor competence were found to be concentrated in the 5th to 15th percentile of motor competence with the most profoundly affected group showing no physical activity detriments. This may indicate that physical activity participation in adulthood is not due to continuing motor difficulties and are instead a continuation of physical activity patterns from childhood. Although not measured in this study, it is also possible that the most severely impaired individuals received more concerted outside effort, such as interventions, to increase their motor skills than the less impaired group, placing them on a more positive physical activity trajectory for adulthood. International studies have found that individuals with more severe motor skill impairment are more likely to show problems, such as handwriting issues, that result in intervention than those with more moderate motor impairments.^1^ This also offers a potential explanation for why physical activity differences found in this study are smaller than what has been reported in previous pediatric accelerometery studies^8^, ^38^. Individuals with DCD may also be less affected by the decrease in MVPA that has been reported to occur in much of the general population in young adulthood.^39^ As a relationship has been demonstrated between decrease in physical activity and reduction in organized physical activity as individuals age,^39^ individuals with DCD may be less affected as they engage less in team and competitive physical activity programs in favor of solitary exercise.^6^ The absence of any significant differences when the DCD risk groups were analyzed by gender may support this theory, as gender‐specific effects reported in other studies have been hypothesized to be due to gender‐specific differences in activity play, sports, and similar physical activities.^5^ Cultural effects, specific to physical activity in Finland,^11^, ^40^ may also be a factor. Studies of Finnish children have found that motor competence did not impact upon cardiorespiratory fitness measures in this population.^41^

Statistical modeling indicated an increased role for DCD risk upon physical activity via its interaction with BMI. Non‐DCD individuals were more affected by BMI changes than the DCD group, with the minutes per day in MVPA decreasing and minutes per day in sedentary light activity increasing at a greater rate as BMI increased. The lesser effect of BMI on physical activity for the DCD group may be due to their physical activity patterns being impacted by their pre‐existing motor competence difficulties and related factors such as avoidance coping strategies, making them less affected by movement difficulties associated with increasing BMI which decreases physical activity in non‐DCD individuals.^42^ Additionally, as movement of individuals with DCD is less efficient, they use more metabolic energy during physical activity.^38^ Hence, individuals with DCD may use the same amount of energy at lower levels of physical activity than is seen in nonaffected individuals such that their BMI reflects the energy efficiency of their movement. The absence of any difference in BMI measurements between DCD risk groups despite physical activity differences would support the idea of differential energy efficiency being a factor in the BMI‐to‐DCD interaction effect, although other casual factors upon BMI were not measured in this study. The differential effect of BMI upon physical activity has not been previously investigated and is an important avenue for further research.

Inefficiency of locomotion effects on BMI cannot be extended to other adverse health outcomes of inactivity. Although no significant differences were found in percentage of participants meeting physical activity guidelines, with MVPA levels being currently sufficient to meet physical activity guidelines, the association of higher levels of sedentary behavior with adverse health outcomes^9^ is worth noting, with the physical activity pattern seen in this study with increased sedentary behavior and decreased vigorous physical activity being particularly detrimental to cardiovascular^43^ and bone health.^44^ Bone health detriments are reported in individuals with DCD, potentially due to a detrimental physical activity pattern.^45^ The current study provides further support for this hypothesis, as although the changes reported in this study are small, with small to medium effect sizes, it is likely that they would result in bone changes, particularly for vigorous physical activity as only a small amount of vigorous physical activity is required to stimulate the formation of bone mineral.^44^ Previous pediatric studies have found a change of −0.5 to –0.7% in bone measurements for every additional hour of sedentary time or reduction of 18 minutes of MVPA,^44^ which if applied to adults in this study could amount to a 0.2 to 0.3 difference in bone measurements, which would be clinically significant on a population level. Further research, directly measuring physical activity levels and bone health in adult DCD populations are required to confirm these findings; however, it may indicate an important area of focus for future research and therapeutic options.

### 4.2 Does VMI impact upon physical activity levels at the age of 25?

Sensitivity analysis of VMI did not show an impact of VMI detriments upon physical activity levels at the 15th percentile level, although lower levels of vigorous physical activity were shown in between group differences at the highest level of detriment (5th percentile). Statistical models including VMI as both a categorical and continuous variable did not show a significant role for VMI in affecting physical activity apart from mean amplitude deviation although risk status for DCD and the DCD‐to‐BMI interaction did become significant in the models for moderate and MVPA. This contrasts with Jarus et al's work in a pediatric population that showed VMI acting as an independent inhibitor of physical activity^16^; however, Jarus's study measured the type of physical activity (i.e., diversity, intensity, and sociality) engaged in rather than total physical activity. This study particularly the significant BMI‐to‐VMI interaction effect for mean amplitude deviation and the increased role for DCD risk status in models including VMI indicate a change in choice of physical activity due to VMI in this population and support Jarus’ findings. Since such physical activity choices would not necessarily affect overall energy expenditure, it is unlikely that these changes would impact upon BMI and body fat and so the current study did not provide an explanation for the previous findings from this population that VMI was linked to increased BMI and body fat percentage in early adulthood,^17^ although it supported the findings in regard to differences in BMI and BMI trajectory based on VMI risk status. Given the higher rate of some medical interventions and neonatal complications in this group, it is possible that VMI reflects differences in development which independently relate to BMI and body fat, as the current study did not find a causal pathway with physical activity, nor does it appear to be via its impact on motor competence. Examination of motor competence scores in this group showed that although the DCD groups showed detriments in VMI scores similar to what has been reported in other studies^14^ the reverse was not the case and VMI as measured by the Beery test, did not offer sufficient sensitivity to be used as a marker for DCD. As such, evidence for VMI’s role in predicting health outcomes was not found and does not appear to be related to its association with DCD or its impact on physical activity.

### 4.3 Strengths and limitations

The longitudinal nature of this study, including follow‐up over a 20‐year period, is a strength as longitudinal measures provide an additional insight into the effects of motor competence on physical activity. This study by measuring motor competence at 5 years and then physical activity in adulthood shows the long‐term implications of impaired motor competence in early life, rather than showing the effects on motor competence of inactivity. This is particularly an issue for studies on motor competence in adulthood as these studies are often cross‐sectional and thus likely to be confounded by the effects of prior experience, BMI, and increased body stature on performance on motor competence test items.^42^ This study did not re‐evaluate motor competence at the age of 25 years; however, most of the group would be anticipated to continue to have motor competence issues into adulthood,^1^ with this study focused only on the effect of childhood low motor competence as is seen in DCD on adulthood physical activity. This study did not assess physical activity at age 5 years and as such it is not known whether the reported physical activity patterns were established in childhood or occurred later in life.

Cross‐cultural issues related to physical activity should be considered in interpreting the results from this study as Finland has a high level of leisure physical activity participation with less reliance on organized sports or structured environments than present in other countries.^11^ Given that adults with DCD report less physical activity in organized sports and structured environments and more exercise that is solitary or with their immediate social group,^6^ a smaller difference in physical activity may be present in this population than is found cross culturally. The type of physical activity was not collected for this study; however, the likely low levels of organized sports participation in the non‐DCD group provide an opportunity to examine leisure‐based physical activity in DCD, which is likely the largest contributor to their physical activity levels. Furthermore, as specific facilitators and barriers to physical activity may be present in different environments and cross‐cultural studies have indicated a cross‐cultural effect on physical activity in DCD,^8^ validation and applicability of these findings in other countries are warranted.

The AYLS cohort is a longitudinal observational study, and as such, causality cannot be assigned. In addition, although many health confounders were examined, confounding by other unmeasured variables is still a risk. A particular concern for confounding is from the effects of attention deficit disorder, particularly the hyperactive form (ADHD), which is commonly comorbid in individuals with DCD,^1^ and for which data were not available for this study. Pediatric studies have shown that children with both DCD and ADHD have a smaller deficit in activity levels compared to their typically developing peers than children with DCD alone,^36^ and so failing to control for this factor may have resulted in underestimating the degree of deficit in activity levels in individuals with DCD.

## 5 PERSPECTIVES

Early DCD risk status was associated with lower levels of physical activity in young adults, providing device measured evidence that deficits in physical activity shown in childhood and adolescence in individuals with DCD extends into adulthood. Childhood DCD status appeared to mediate the role of BMI upon physical activity, such that individuals with DCD did not show as much decrease in physical activity with increasing BMI, potentially due to higher energy requirements for movement in individuals with DCD. However, the physical activity pattern demonstrated if continued through the lifespan is likely to place this population at an increased risk of sedentary related adverse health outcomes and highlights a continued need for physical activity interventions to improve physical activity into adulthood.

## CONFLICT OF INTEREST

The authors declare no conflicts of interest. The results of the study are presented clearly, honestly and without fabrication, falsification, or inappropriate data manipulation.

## AUTHOR CONTRIBUTIONs

JT is responsible for statistical analysis of the data and prepared the first draft of the paper. Authors PC, TR, and NH contributed to the analysis. AL participated in the AYLS childhood data collection and assessments, AL and JE in conceptualizing the AYLS adulthood data collection, MS and NW participated in accelerometery data collection and AYK contributed childhood data. HP conceptualized and designed this study, analyzed the data, and interpreted the results. All authors critically reviewed the manuscript for important intellectual content.

## Supporting information


Appendix A

Appendix B

Appendix C

Appendix D

Appendix E‐1

Appendix E‐2

Appendix F

Appendix G
