# Supplementary material for: Effect of childhood developmental coordination disorder on adulthood physical activity; Arvo Ylppö longitudinal study
Source: Scand J Med Sci Sports. 2022 Feb 24;32(6):1050–63. doi: 10.1111/sms.14144 (PMC9306991; doi:10.1111/sms.14144)
Supplement: Supplementary file 1 — Appendix A [file SMS-32-1050-s002.docx]

## **Appendix A**

Motor competence items and anomality criteria

| **Item** | **Criteria for anormality** |
| --- | --- |
| Pursing the lips to whistle | Not achieved |
| Tongue position maintained when stuck out | Not achieved |
| Imitates tongue movements | Performed with difficulty or not possible |
| Running | Only possible when pace was slow |
| Going upstairs | Only achieved with support or performed with even pace |
| Sideways hopping | Performed less than two jumps |
| Standing on one leg (right/left) | Performed for less than 5 seconds |
| Hopping on one leg | Performed for less than five seconds |
| Forefinger-thumb tapping (right/left) | Could not achieve or achieved only in a jerky fashion |
| Finger-opposition (right/left) | Not possible |
| Pegboard (right/left) | Speed of performance in bottom 5% of control population |
| Hand pro-supination (R/L) | Unable to perform with rhythm |
| Simultaneous handclapping | Unable to perform with rhythm |
| Alternate handclapping | Unable to perform with rhythm |

Item list derived from Lano (23)
