# Supplementary material for: Effect of childhood developmental coordination disorder on adulthood physical activity; Arvo Ylppö longitudinal study
Source: Scand J Med Sci Sports. 2022 Feb 24;32(6):1050–63. doi: 10.1111/sms.14144 (PMC9306991; doi:10.1111/sms.14144)
Supplement: Supplementary file 2 — Appendix B [file SMS-32-1050-s007.docx]

## **Appendix B Background characteristics of participants according to VMI status**

|  | VMI <5^th^ percentile | VMI 5^th^ to 15^th^ percentile | VMI >15^th^ percentile | Group difference | |
| --- | --- | --- | --- | --- | --- |
| **Parental characteristics** | | | | | |
|  | *M (SD) [Md]* | *M (SD) [Md]* | *M (SD) [Md]* | *H statistic* | *p* |
| Mother’s age (yrs) | 28.9 (5.9) [28.0] | 29.1 (4.7) [29.0] | 30.2 (4.8) [30.0] | 2.4 | .308 |
| Father’s age (yrs) | 31.9 (5.6) [32.0] | 32.0 (6.2) [31.0] | 32.3 (5.9)[31.0] | 0.3 | .882 |
|  | *n (%)* | *n (%)* | *n (%)* | *χ^2^* | *p* |
| Mother’s education – primary only | 40.9 | 18.8 | 17.9 | 9.8 | .132 |
| Mother’s education – secondary only | 18.2 | 18.8 | 20.3 |  |  |
| Mother’s education -upper secondary only | 27.3 | 53.1 | 43.1 |  |  |
| Mother’s education – Masters | 13.6 | 9.4 | 18.8 |  |  |
| Father’s education- primary only | 40.9 | 46.9 | 18.0 | 29.2 | <.001 |
| Father’s education – secondary only | 45.5 | 21.9 | 29.0 |  |  |
| Father’s education- upper secondary only | 4.5 | 12.5 | 27.7 |  |  |
| Father’s education- Master’s | 9.1 | 18.8 | 25.3 |  |  |
| **Pre and perinatal risk factors** | | | | | |
|  | *n (%)* | *n (%)* | *n (%)* | *χ^2^* | *p* |
| Mother severe chronic illness | 0.0 | 3.1 | 6.4 | 2.1 | .353 |
| Multiple pregnancy | 8.7 | 9.4 | 4.3 | 2.5 | .284 |
| Pre-eclampsia | 8.7 | 12.5 | 12.4 | 0.3 | .868 |
| Fetal distress during pregnancy | 0.9 | 6.3 | 6.9 | 0.1 | .934 |
| Fetal distress during birth | 21.7 | 18.8 | 16.0 | 0.7 | .714 |
|  | *M (SD) [Md]* | *M (SD) [Md]* | *M (SD) [Md]* | *H statistic* | *p* |
| Apgar at 5 minutes | 9.4 (1.3) [10.0] | 9.0 (1.3) [9.0] | 9.4 (1.2) [10.0] | 8.2 | .017 |
| **Neonatal characteristics** | |  |  |  |  |
| Sex (m/f) | 16/7 | 18/14 | 272/309 | 5.5 | .065 |
|  | *M (SD) [Md]* | *M (SD) [Md]* | *M (SD) [Md]* | *H statistic* | *p* |
| Gestational age (wks) | 38.0 (3.4) [39.0] | 38.0 (3.1) [39.0] | 38.7 (2.5) [39.0] | 2.5 | .285 |
| Birth Weight (g) | 3370.4 (838.0) [3530.0] | 3076.3 (720.7) [3190.0] | 3396.5 (721.7) [3490.0] | 6.4 | .041 |
|  | *n (%)* | *n (%)* | *n (%)* | *χ^2^* | *p* |
| Small for gestational age (less than 2 SD below mean) | 4.3 | 6.3 | 6.0 | 0.1 | .943 |
| **Neonatal risk factors/complications** | | | | | |
|  | *n (%)* | *n (%)* | *n (%)* | *χ^2^* | *p* |
| Hospitalized | 60.8 | 62.5 | 62.8 | 0.04 | .982 |
| Intubation or ventilator treatment | 8.7 | 15.6 | 9.1 | 1.5 | .469 |
| Suspicion/verified of septic infection | 8.7 | 3.1 | 5.7 | 0.8 | .676 |
| Surgical operation | 4.3 | 6.3 | 1.0 | 7.4 | .025 |
| Severe anemia requiring blood transfusion | 8.7 | 3.1 | 4.1 | 1.2 | .538 |
| Apnea | 8.7 | 8.3 | 2.1 | 9.6 | .008 |
| Clinical seizures | 4.3 | 9.4 | 0.9 | 17.2 | <.001 |
| IVH grade 1-2 | 8.7 | 3.1 | 0.7 | 14.2 | .001 |
| **Characteristics at 56 months follow up** | | | | | |
|  | *M (SD) [Md]* | *M (SD) [Md]* | *M (SD) [Md]* | *H statistic* | *p* |
| Age | 4.68 (0.02) [4.68] | 4.71 (0.04) [4.71] | 4.71 (0.04) [4.71] | 14.7 | .001 |
| Weight | 17.9 (2.2) [18.0] | 18.4 (3.1) [18.1] | 18.2 (2.5) [18.0] | 0.2 | .912 |
| Height | 107.4 (3.5) [108.0] | 108.7 (4.6) [108.0] | 108.4 (4.5) [108.0] | 0.9 | .638 |
| BMI | 15.4 (1.3) [15.2] | 15.5 (1.6) [15.1] | 15.5 (1.4) [15.4] | 0.6 | .727 |
| Motor competence (%) sum score | 98.2 (4.0) [100.0] | 99.0 (2.3) [100.0] | 99.3 (2.5) [100.0] | 5.0 | .083 |
| Gross motor (Touwen)(% sum score) | 94.9 (11.7) [100.0] | 93.8 (13.2) [100.0] | 95.7 (10.5) [100.0] | 0.4 | .829 |
| Fine motor (Touwen)(% sum score) | 63.8 (30.0) [66.7] | 75.0 (26.8) [66.7] | 89.4 (18.8) [100.0] | 37.1 | <.001 |
| VMI score | 72.8 (5.1) [75.5] | 76.9 (2.8) [75.6] | 103.5 (12.2) [103.9] | 150.8 | <.001 |
|  | *n (%)* | *n (%)* | *n (%)* | *χ^2^* | *p* |
| Eyesight 0.3-0.6 | 4.4 | 15.6 | 14.0 | 1.8 | .399 |
| BMI grouping - underweight | 0.0 | 0.0 | 1.8 | 7.1 | .308 |
| BMI grouping – healthy weight | 72.7 | 84.4 | 83.0 |  |  |
| BMI grouping - overweight | 27.3 | 9.4 | 12.6 |  |  |
| BMI grouping-obese | 0.0 | 6.3 | 2.7 |  |  |
| Abnormal gross motor | 13.0 | 12.5 | 6.1 | 3.6 | .165 |
| Abnormal fine motor | 34.8 | 15.6 | 5.0 | 36.1 | <.001 |
| Unable to ride a bike | 0.0 | 0.0 | 1.7 | 1.0 | .617 |
| Hardly able to catch a ball | 17.4 | 21.4 | 16.0 | 0.6 | .740 |
| Only able to run slowly | 0.0 | 3.1 | 3.8 | 0.9 | .626 |
| **Characteristics at 25 year follow up** | | | | | |
|  | *M (SD) [Md]* | *M (SD) [Md]* | *M (SD) [Md]* | *H statistic* | *p* |
| Age | 25.3 (0.8) [25.0] | 24.8 (0.5) [25.0] | 24.8 (0.7) [25.0] | 7.4 | .025 |
| Weight | 79.5 (17.8) [78.5] | 71.2 (18.9) [66.7] | 72.2 (15.0) [70.3] | 5.4 | .067 |
| Height | 174.6 (9.8) [178.0] | 174.1 (8.9) [175.0] | 173.0 (9.5) [173.0] | 1.8 | .415 |
| BMI | 26.0 (5.1) [24.9] | 23.2 (4.7) [22.7] | 24.0 (4.2) [23.2] | 6.4 | .041 |
|  | *n (%)* | *n (%)* | *n (%)* | *χ^2^* | *p* |
| BMI grouping-underweight | 4.5 | 12.5 | 3.1 | 11.6 | .071 |
| BMI grouping – healthy weight | 45.5 | 62.5 | 64.3 |  |  |
| BMI grouping - overweight | 31.8 | 18.8 | 23.3 |  |  |
| BMI grouping - obese | 18.2 | 6.3 | 9.2 |  |  |
| **Quantified physical activity** | | | | | |
|  | *M (SD) [Md]* | *M (SD) [Md]* | *M (SD) [Md]* | *H statistic* | *p* |
| Sedentary Light | 873.1 (91.7) [895.9] | 831.8 (101.6) [809.0] | 837.9 (106.8) [856.9] | 3.0 | .227 |
| Moderate | 123.5 (61.0) [111.4] | 163.9 (80.1) [156.9] | 137.8 (79.0) [121.2] | 4.5 | .105 |
| Vigorous | 4.6 (8.6) [1.1] | 8.1 (9.6) [6.6] | 6.6 (8.1) [3.8] | 6.0 | .051 |
| Moderate and vigorous | 128.1 (67.3) [113.3] | 171.9 (84.9) [160.8] | 144.4 (82.3) [127.9] | 4.6 | .098 |
| % sedentary light activity | 63.3 (5.6) [63.9] | 60.5 (7.0) [59.7] | 61.3 (6.5) [61.9] | 3.1 | .215 |
| % moderate activity | 8.9 (4.3) [8.0] | 11.9 (5.7) [11.8] | 10.1 (5.7) [8.8] | 4.5 | .104 |
| % vigorous activity | 0.3 (0.6) [0.1] | 0.6 (0.7) [0.3] | 0.5 (0.6) [0.3] | 6.0 | .049 |
| % moderate and vigorous activity | 9.2 (4.8) [8.1] | 12.5 (6.1) [12.1] | 10.6 (6.0) [9.3] | 4.8 | .092 |
| Steps | 9842.1 (3317.0) [9705.5] | 10858.0 (4114.4) [10418.3] | 10208.7 (3634.4) [9827.6] | 0.7 | .707 |
| Mean amplitude deviation | 0.99 (0.2) [0.96] | 1.1 (0.3) [0.96] | 0.98 (0.3) [0.96] | 1.2 | .541 |
