# Supplementary material for: Effect of childhood developmental coordination disorder on adulthood physical activity; Arvo Ylppö longitudinal study
Source: Scand J Med Sci Sports. 2022 Feb 24;32(6):1050–63. doi: 10.1111/sms.14144 (PMC9306991; doi:10.1111/sms.14144)
Supplement: Supplementary file 3 — Appendix C [file SMS-32-1050-s005.docx]

## Appendix C

## Accelerometry complex models for physical activity with DCD as a probable risk factor

|  |  |  |  | β 95% Confidence interval | |  |
| --- | --- | --- | --- | --- | --- | --- |
| Model |  | β | S.E. | Lower | Upper | P |
| Sedentary light | Intercept | 6.5 | 0.03 | 6.4 | 6.6 | <.001 |
|  | Sex^†^ | -0.01 | 0.01 | -0.03 | 0.01 | .544 |
|  | Mother’s education (secondary)^§^ | -0.01 | 0.02 | -0.04 | 0.03 | .645 |
|  | Mother’s education (upper secondary) ^¶^ | -0.003 | 0.02 | -0.04 | 0.03 | .845 |
|  | Mother’s education (Masters)^††^ | 0.001 | 0.01 | -0.03 | 0.03 | .932 |
|  | DCD^‡^ | 0.1 | 0.06 | 0.02 | 0.3 | .027 |
|  | BMI | 0.01 | 0.001 | 0.007 | 0.01 | <.001 |
|  | BMI*DCD interaction effect | -0.01 | 0.003 | -0.01 | 0.0 | .057 |
| Moderate | Intercept | 6.4 | 0.1 | 6.2 | 6.7 | <.001 |
|  | Sex^†^ | 0.2 | 0.04 | 0.1 | 0.3 | <.001 |
|  | Mother’s education (secondary)^§^ | 0.01 | 0.1 | -0.1 | 0.1 | .869 |
|  | Mother’s education (upper secondary) ^¶^ | 0.03 | 0.1 | -0.1 | 0.2 | .586 |
|  | Mother’s education (Masters)^††^ | 0.05 | 0.1 | -0.1 | 0.1 | .366 |
|  | DCD^‡^ | -0.5 | 0.3 | -1.1 | 0.1 | .073 |
|  | BMI | -0.1 | 0.01 | -0.08 | -0.06 | <.001 |
|  | BMI* DCD interaction effect | 0.02 | 0.01 | 0.0 | 0.04 | .102 |
| Vigorous | Intercept | 3.5 | 0.2 | 3.1 | 3.9 | <.001 |
|  | Sex^†^ | 0.1 | 0.1 | -0.03 | 0.3 | .107 |
|  | Mother’s education (secondary)^§^ | -0.2 | 0.1 | -0.4 | 0.1 | .223 |
|  | Mother’s education (upper secondary) ^¶^ | -0.1 | 0.1 | -0.3 | 0.2 | .545 |
|  | Mother’s education (Masters)^††^ | 0.03 | 0.1 | -0.2 | 0.2 | .776 |
|  | DCD^‡^ | -0.9 | 0.5 | -1.8 | 0.04 | .062 |
|  | BMI | -0.1 | 0.01 | -0.1 | -0.07 | <.001 |
|  | BMI* DCD interaction effect | 0.04 | 0.02 | 0.001 | 0.1 | .050 |
| MVPA | Intercept | 6.5 | 0.1 | 6.3 | 6.8 | <.001 |
|  | Sex^†^ | 0.2 | 0.04 | 0.1 | 0.3 | <.001 |
|  | Mother’s education (secondary)^§^ | 0.002 | 0.1 | -0.1 | 0.1 | .971 |
|  | Mother’s education (upper secondary) ^¶^ | 0.02 | 0.1 | -0.1 | 0.1 | .716 |
|  | Mother’s education (Masters)^††^ | 0.04 | 0.1 | -0.1 | 0.1 | .397 |
|  | DCD^‡^ | -0.5 | 0.3 | -1.1 | 0.0 | .053 |
|  | BMI | -0.1 | 0.01 | -0.09 | -0.06 | <.001 |
|  | BMI* DCD | 0.02 | 0.01 | -0.002 | 0.04 | .076 |
| Steps | Intercept | 9.6 | 0.1 | 9.4 | 9.8 | <.001 |
|  | Sex^†^ | -0.1 | 0.03 | -0.2 | 0.1 | <.001 |
|  | Mother’s education (secondary)^§^ | -0.01 | 0.05 | -0.1 | 0.1 | .776 |
|  | Mother’s education (upper secondary) ^¶^ | -0.04 | 0.04 | -0.1 | 0.04 | .307 |
|  | Mother’s education (Masters)^††^ | 0.03 | 0.04 | -0.1 | 0.1 | .459 |
|  | DCD^‡^ | -0.2 | 0.2 | -0.6 | 0.2 | .270 |
|  | BMI | -0.01 | 0.004 | -0.02 | -0.01 | <.001 |
|  | BMI* DCD interaction effect | 0.01 | 0.007 | -0.01 | 0.02 | .386 |
| Mean amplitude deviation | Intercept | 0.4 | 0.1 | 0.2 | 0.5 | <.001 |
|  | Sex^†^ | -0.004 | 0.02 | -0.04 | 0.04 | .851 |
|  | Mothers education (secondary)^§^ | 0.01 | 0.03 | -0.05 | 0.1 | .672 |
|  | Mothers education (upper secondary) ^¶^ | 0.01 | 0.03 | -0.1 | 0.1 | .818 |
|  | Mothers education (Masters)^††^ | 0.01 | 0.03 | -0.04 | 0.1 | .615 |
|  | DCD^‡^ | -0.04 | 0.1 | -0.3 | 0.3 | .801 |
|  | BMI | -0.02 | 0.003 | -0.02 | -0.01 | <.001 |
|  | BMI* DCD interaction effect | 0.001 | 0.01 | -0.01 | 0.01 | .857 |

† Where male is the comparison group and β=1; ‡ Where DCD is the comparison group and β=1; § Where education is level 1; ¶ Where education is level 2; †† Where education is level 3
