# Supplementary material for: Effect of childhood developmental coordination disorder on adulthood physical activity; Arvo Ylppö longitudinal study
Source: Scand J Med Sci Sports. 2022 Feb 24;32(6):1050–63. doi: 10.1111/sms.14144 (PMC9306991; doi:10.1111/sms.14144)
Supplement: Supplementary file 4 — Appendix D [file SMS-32-1050-s004.docx]

## Appendix D

## Accelerometry complex models for physical activity with DCD (DCD5 and DCD15) and VMI as a continuous variable

|  |  |  |  | β 95% Confidence interval | |  |
| --- | --- | --- | --- | --- | --- | --- |
| Model |  | β | S.E. | Lower | Upper | P |
| Sedentary light | Intercept | 6.5 | 0.1 | 6.4 | 6.5 | <.001 |
|  | Sex^†^ | -0.01 | 0.01 | -0.03 | 0.01 | .503 |
|  | Mother’s education (secondary)^§^ | -0.01 | 0.02 | -0.1 | 0.03 | .633 |
|  | Mother’s education (upper secondary)^¶^ | -0.001 | 0.02 | -0.03 | 0.03 | .952 |
|  | Mother’s education (Masters)^††^ | 0.004 | 0.02 | -0.03 | 0.03 | .783 |
|  | DCD^‡^ | 0.2 | 0.1 | 0.1 | 0.3 | .007 |
|  | VMI score | 0.0 | 0.0004 | 0.0 | 0.001 | .300 |
|  | BMI | 0.01 | 0.001 | 0.007 | 0.01 | <.001 |
|  | BMI* DCD interaction | -0.01 | 0.003 | -0.01 | -0.001 | .019 |
| Moderate | Intercept | 6.7 | 0.2 | 6.3 | 7.04 | <.001 |
|  | Sex^†^ | 0.2 | 0.04 | 0.1 | 0.3 | <.001 |
|  | Mother’s education (secondary)^§^ | 0.02 | 0.1 | -0.1 | 0.1 | .714 |
|  | Mother’s education (upper secondary)^¶^ | 0.02 | 0.1 | -0.1 | 0.1 | .708 |
|  | Mother’s education (Masters)^††^ | 0.03 | 0.1 | -0.1 | 0.1 | .548 |
|  | DCD^‡^ | -0.6 | 0.3 | -1.2 | -0.1 | .020 |
|  | VMI score | -0.002 | 0.002 | -0.01 | 0.001 | .176 |
|  | BMI | -0.1 | 0.01 | -0.08 | -0.06 | <.001 |
|  | BMI* DCD interaction | 0.03 | 0.01 | 0.0 | 0.05 | .027 |
| Vigorous | Intercept | 3.2 | 0.4 | 2.5 | 4.0 | <.001 |
|  | Sex^†^ | 0.1 | 0.08 | -0.01 | 0.3 | .072 |
|  | Mother’s education (secondary)^§^ | -0.2 | 0.1 | -0.4 | 0.1 | .183 |
|  | Mother’s education (upper secondary)^¶^ | -0.1 | 0.1 | -0.3 | 0.2 | .615 |
|  | Mother’s education (Masters)^††^ | 0.03 | 0.1 | -0.2 | 0.2 | .775 |
|  | DCD^‡^ | -0.8 | 0.5 | -1.7 | 0.2 | .137 |
|  | VMI score | 0.003 | 0.003 | 0.0 | 0.01 | .256 |
|  | BMI | -0.1 | 0.01 | -0.1 | -0.07 | <.001 |
|  | BMI* DCD interaction | 0.03 | 0.02 | 0.0 | 0.1 | .078 |
| MVPA | Intercept | 6.7 | 0.2 | 6.3 | 7.1 | <.001 |
|  | Sex^†^ | 0.2 | 0.04 | 0.1 | 0.3 | <.001 |
|  | Mother’s education (secondary)^§^ | 0.01 | 0.1 | -0.1 | 0.1 | .835 |
|  | Mother’s education (upper secondary)^¶^ | 0.01 | 0.1 | -0.1 | 0.1 | .835 |
|  | Mother’s education (Masters)^††^ | 0.03 | 0.1 | -0.1 | 0.1 | .580 |
|  | DCD^‡^ | -0.7 | 0.3 | -1.2 | -0.1 | .014 |
|  | VMI score | -0.002 | 0.002 | -0.01 | 0.0 | .258 |
|  | BMI | -0.08 | 0.01 | -0.09 | -0.07 | <.001 |
|  | BMI*DCD interaction | 0.03 | 0.01 | 0.0 | 0.05 | .018 |
| Steps | Intercept | 9.6 | 0.1 | 9.4 | 9.9 | <.001 |
|  | Sex^†^ | -0.1 | 0.03 | -0.2 | -0.1 | <.001 |
|  | Mother’s education (secondary)^§^ | -0.01 | 0.05 | -0.1 | 0.1 | .800 |
|  | Mother’s education (upper secondary)^¶^ | -0.05 | 0.04 | -0.1 | 0.03 | .252 |
|  | Mother’s education (Masters)^††^ | 0.02 | 0.04 | -0.1 | 0.1 | .640 |
|  | DCD^‡^ | -0.3 | 0.2 | -0.6 | 0.1 | .204 |
|  | VMI score | -0.001 | 0.001 | -0.003 | 0.002 | .609 |
|  | BMI | -0.02 | 0.004 | -0.02 | -0.01 | <.001 |
|  | BMI*DCD interaction | 0.01 | 0.01 | -0.01 | 0.02 | .252 |
| Mean amplitude deviation | Intercept | 0.5 | 0.1 | 0.3 | 0.7 | <.001 |
|  | Sex^†^ | -0.01 | 0.02 | -0.1 | 0.0 | .734 |
|  | Mother’s education (secondary)^§^ | 0.01 | 0.03 | -0.1 | 0.1 | .743 |
|  | Mother’s education (upper secondary)^¶^ | -0.001 | 0.03 | -0.1 | 0.1 | .970 |
|  | Mother’s education (Masters)^††^ | 0.01 | 0.03 | 0.0 | 0.1 | .766 |
|  | DCD^‡^ | -0.1 | 0.2 | -0.4 | 0.2 | .718 |
|  | VMI score | -0.001 | 0.001 | -0.002 | 0.001 | .315 |
|  | BMI | -0.02 | 0.003 | -0.02 | -0.01 | <.001 |
|  | BMI*DCD interaction | 0.002 | 0.01 | -0.01 | 0.01 | .748 |

† Where male is the comparison group and β=1; ‡ Where DCD is the comparison group and β=1; § Where education is level 1; ¶ Where education is level 2; †† Where education is level 3
