# Supplementary material for: Effect of childhood developmental coordination disorder on adulthood physical activity; Arvo Ylppö longitudinal study
Source: Scand J Med Sci Sports. 2022 Feb 24;32(6):1050–63. doi: 10.1111/sms.14144 (PMC9306991; doi:10.1111/sms.14144)

## **Appendix E**

Between group difference tests showed no significant difference in accelerometry measures at the 15^th^ percentile and a significant difference in vigorous physical activity at the 5^th^ percentile (Table 5), with those in the 5^th^ percentile performing significantly less vigorous physical activity than those above the 15^th^ percentile.

Modelling of the bottom 15^th^ percentile of VMI scores did not show significant role for VMI risk (Table 6), with BMI being the only statistically significant influencer in all models, with sex being an influencer for the models for moderate activity, MVPA and steps, and an interaction effect between BMI and the VMI risk group in the model for mean MAD. These are shown in table eleven. This interaction effect was such that mean MAD decreased with increasing BMI for the VMI risk group only (Figure 3).

### **Table 5: Accelerometry differences based on VMI risk status (5^th^ percentile)**

|  | Less than 5%  N=23 | Greater than 15%  N=581 | Group difference | |  |
| --- | --- | --- | --- | --- | --- |
|  | *M (SD) [Md]* | *M (SD) [Md]* | ***d_Cohen_*** | U-statistic | P |
| Age | 25.3 (0.8) [25.0] | 24.8 (0.7) [25.0] | -0.7 | 2.8 ^†^ | .005 |
| BMI | 26.0 (5.1) [24.9] | 24.0 (4.2) [23.2] | 0.5 | 4919.0 | .032 |
| Sedentary light (min/day) | 873.1 (91.7) [895.9] | 837.9 (106.8) [856.9] | -0.3 | 5485.0 | .145 |
| Moderate (min/day) | 123.5 (61.0) [111.4] | 137.8 (79.0) [121.2] | 0.2 | 6192.0 | .551 |
| Vigorous (min/day) | 4.6 (8.6) [1.1] | 6.6 (8.1) [3.8] | 0.2 | 4780.0 | .021 |
| Moderate and vigorous (min/day) | 128.1 (67.3) [113.3] | 144.4 (82.3) [127.9] | 0.2 | 6017.5 | .419 |
| % sedentary light activity | 63.3 (5.6) [63.9] | 61.3 (6.4) [61.9] | -0.3 | 5701.0 | .119 |
| % moderate activity | 8.9 (4.3) [8.0] | 10.1 (5.7) [8.8] | 0.1 | 6332.0 | .407 |
| % vigorous activity | 0.3 (0.6) [0.1] | 0.5 (0.6) [0.3] | 0.2 | 4997.0 | .018 |
| % moderate and vigorous activity | 9.2 (4.8) [8.1] | 10.6 (6.0) [9.3] | 0.2 | 6170.0 | .309 |
| Steps | 9842.1 (3317.0) [9705.5] | 10208.7 (3634.4) [9827.6] | 0.1 | 6390.0 | .722 |
| Mean amplitude deviation | 1.0 (0.2) [1.0] | 1.0 (0.3) [1.0] | 0.0 | 6555.0 | .878 |
|  | 5-15%  N=32 | Greater than 15%  N=581 | Group difference | | |
|  | *M (SD) [Md]* | *M (SD) [Md]* | ***d_Cohen_*** | U-statistic | P |
| Age (yrs) | 24.8 (0.5) [25.0] | 24.8 (0.7) [25.0] | 0.0 | -0.1^†^ | .959 |
| BMI | 23.2 (4.7) [22.7] | 24.0 (4.2) [23.2] | 0.2 | 8069.0 | .208 |
| Sedentary light (min/day) | 831.8 (101.6) [809.0] | 837.9 (106.8) [856.9] | 0.2 | 8435.5 | .378 |
| Moderate (min/day) | 163.9 (80.1) [156.9] | 137.8 (79.0) [121.2] | -0.3 | 7349.0 | .046 |
| Vigorous (min/day) | 8.1 (9.6) [4.4] | 6.6 (8.1) [3.8] | -0.2 | 8610.5 | .482 |
| Moderate and vigorous (min/day) | 171.9 (84.9) [160.8] | 144.4 (82.3) [127.9] | -0.3 | 7385.0 | .050 |
| % sedentary light activity | 60.5 (7.0) [59.7] | 61.3 (6.4) [62.0] | 0.1 | 8493.0 | .410 |
| % moderate activity | 11.9 (5.7) [11.8] | 10.1 (5.7) [8.8] | -0.3 | 7391.0 | .051 |
| % vigorous activity | 0.6 (0.7) [0.3] | 0.5 (0.6) [0.3] | -0.2 | 8658.0 | .513 |
| % moderate and vigorous activity | 12.5 (6.1) [12.1] | 10.6 (6.0) [9.3] | -0.3 | 7411.0 | .053 |
| Steps | 10858.0 (4114.4) [10418.3] | 10208.7 (3634.4) [9827.6] | -0.2 | 8577.0 | .461 |
| Mean amplitude deviation | 1.1 (0.3) [1.0] | 1.0 (0.3) [1.0] | -0.3 | 8221.0 | .270 |
|  | Less than 15%  N=55 | Greater than 15%  N=581 | Group difference | | |
|  | *M (SD) [Md]* | *M (SD) [Md]* | ***d_Cohen_*** | U statistic | p |
| Age (yrs) | 25.0 (0.7) [25.0] | 24.8 (0.7) [25.0] | -0.2 | 1.8^†^ | .071 |
| BMI | 24.4 (5.0) [23.5] | 24.0 (4.2) [23.2] | -0.1 | 15442.0 | .681 |
| Sedentary light (min/day) | 849.1 (98.9) [831.5] | 837.9 (106.8) [856.9] | -0.1 | 15641.5 | .796 |
| Moderate (min/day) | 147.0 (74.9) [133.1] | 137.8 (79.0) [121.2] | -0.1 | 14520.0 | .263 |
| Vigorous (min/day) | 6.6 (9.3) [2.7] | 6.6 (8.1) [3.8] | -0.002 | 14761.5 | .350 |
| Moderate and vigorous (min/day) | 153.6 (80.4) [135.9] | 144.4 (82.3) [127.9] | -0.1 | 14730.5 | .338 |
| % sedentary light activity | 61.4 (5.6) [63.9] | 61.3 (6.4) [61.9] | -0.04 | 15514.0 | .722 |
| % moderate activity | 8.9 (4.3) [8.0] | 10.1 (5.7) [8.8] | -0.1 | 14685.0 | .317 |
| % vigorous activity | 0.3 (0.6) [0.1] | 0.5 (0.6) [0.3] | 0.0 | 14685.0 | .321 |
| % moderate and vigorous activity | 9.2 (4.8) [8.1] | 10.6 (6.0) [9.3] | -0.1 | 14855.0 | .389 |
| Steps | 10433.2 (3802.2) [9705.5] | 10208.7 (3634.4) [9827.6] | -0.1 | 15550.0 | .743 |
| Mean amplitude deviation | 1.03 (0.3) [0.96] | 0.99 (0.3) [0.96] | -0.2 | 14776.0 | .356 |

†=t-test

### **Table 6: Accelerometry complex models for physical activity for VMI categorised on the 15th percentile**

|  |  |  |  | 95% Confidence interval | |  |
| --- | --- | --- | --- | --- | --- | --- |
| Model |  | β | S.E. | Lower | Upper | P |
| Sedentary light | Intercept | 6.5 | 0.03 | 6.5 | 6.6 | <.001 |
|  | Sex^†^ | -0.01 | 0.01 | -0.03 | 0.02 | .645 |
|  | Mother’s education (secondary)^§^ | -0.01 | 0.02 | -0.05 | 0.03 | .562 |
|  | Mother’s education (upper secondary)^¶^ | -0.002 | 0.02 | -0.04 | 0.03 | .902 |
|  | Mother’s education (Masters)^††^ | 0.004 | 0.02 | -0.03 | 0.03 | .770 |
|  | VMI category^‡^ | -0.01 | 0.1 | -0.1 | 0.1 | .882 |
|  | BMI | 0.01 | 0.001 | 0.006 | 0.01 | <.001 |
|  | BMI*VMI interaction | 0.001 | 0.003 | -0.004 | 0.01 | .713 |
| Moderate | Intercept | 6.4 | 0.1 | 6.1 | 6.6 | <.001 |
|  | Sex^†^ | 0.2 | 0.04 | 0.1 | 0.3 | <.001 |
|  | Mother’s education (secondary)^§^ | 0.02 | 0.06 | -0.1 | 0.1 | .713 |
|  | Mother’s education (upper secondary)^¶^ | 0.02 | 0.06 | -0.1 | 0.1 | .785 |
|  | Mother’s education (Masters)^††^ | 0.03 | 0.05 | -0.1 | 0.1 | .581 |
|  | VMI category^‡^ | -0.3 | 0.3 | -0.8 | 0.2 | .271 |
|  | BMI | -0.07 | 0.01 | -0.08 | -0.06 | <.001 |
|  | BMI*VMI interaction | 0.02 | 0.01 | -0.01 | 0.04 | .177 |
| Vigorous | Intercept | 3.4 | 0.2 | 2.9 | 3.8 | <.001 |
|  | Sex^†^ | 0.1 | 0.1 | -0.01 | 0.3 | .069 |
|  | Mother’s education (secondary)^§^ | -0.2 | 0.1 | -0.4 | 0.1 | .130 |
|  | Mother’s education (upper secondary)^¶^ | -0.1 | 0.1 | -0.3 | 0.2 | .474 |
|  | Mother’s education (Masters)^††^ | 0.02 | 0.1 | -0.2 | 0.2 | .882 |
|  | VMI category^‡^ | 0.2 | 0.5 | -0.9 | 1.3 | .710 |
|  | BMI | -0.08 | 0.01 | -0.1 | -0.06 | <.001 |
|  | BMI*VMI interaction | -0.01 | 0.02 | -0.05 | 0.03 | .560 |
| MVPA | Intercept | 6.5 | 0.1 | 6.2 | 6.7 | <.001 |
|  | Sex^†^ | 0.2 | 0.04 | 0.1 | 0.3 | <.001 |
|  | Mothers education (secondary)^§^ | 0.01 | 0.06 | -0.1 | 0.1 | .855 |
|  | Mothers education (upper secondary)^¶^ | 0.01 | 0.06 | -0.1 | 0.1 | .931 |
|  | Mothers education (Masters)^††^ | 0.03 | 0.05 | -0.1 | 0.1 | .627 |
|  | VMI category^‡^ | -0.3 | 0.3 | -0.8 | 0.3 | .310 |
|  | BMI | -0.07 | 0.01 | -0.08 | -0.06 | <.001 |
|  | BMI*VMI interaction | 0.02 | 0.01 | -0.01 | 0.04 | .215 |
| Steps | Intercept | 9.6 | 0.1 | 9.4 | 9.8 | <.001 |
|  | Sex^†^ | -0.1 | 0.03 | -0.2 | -0.08 | <.001 |
|  | Mother’s education (secondary)^§^ | -0.01 | 0.05 | -0.1 | 0.1 | .778 |
|  | Mother’s education (upper secondary)^¶^ | -0.05 | 0.04 | -0.1 | 0.03 | .220 |
|  | Mother’s education (Masters)^††^ | 0.02 | 0.04 | -0.1 | 0.1 | .663 |
|  | VMI category^‡^ | -0.3 | 0.2 | -0.7 | 0.2 | .258 |
|  | BMI | -0.01 | 0.004 | -0.02 | -0.01 | <.001 |
|  | BMI*VMI interaction | 0.01 | 0.01 | -0.01 | 0.03 | .168 |
| Mean amplitude deviation | Intercept | 0.4 | 0.1 | 0.3 | 0.5 | <.001 |
|  | Sex^†^ | -0.01 | 0.02 | -0.05 | 0.03 | .522 |
|  | Mother’s education (secondary)^§^ | 0.01 | 0.03 | -0.1 | 0.1 | .722 |
|  | Mother’s education (upper secondary)^¶^ | -0.004 | 0.03 | -0.06 | 0.06 | .886 |
|  | Mother’s education (Masters)^††^ | 0.01 | 0.03 | -0.04 | 0.06 | .717 |
|  | VMI category^‡^ | 0.2 | 0.2 | -0.5 | 0.05 | .104 |
|  | BMI | -0.02 | 0.003 | -0.02 | -0.01 | <.001 |
|  | BMI*VMI interaction | 0.01 | 0.01 | 0.0 | 0.02 | .034 |

† Where male is the comparison group and β=1; ‡ Where VMI under 15^th^ percentile is the comparison group and β=1; § Where education is level 1; ¶ Where education is level 2; †† Where education is level 3

### **Figure 3: Interaction effect for VMI models for mean amplitude deviation**


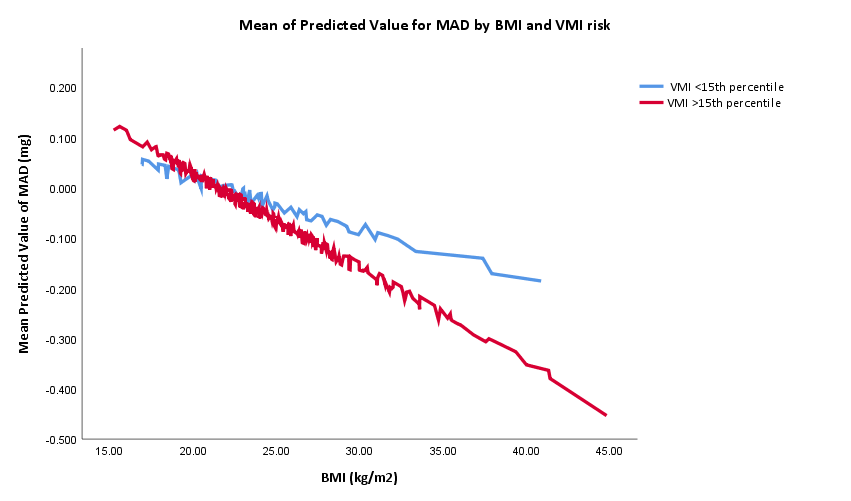

Supplement: Supplementary file 6 — Appendix E‐2 [file SMS-32-1050-s001.docx]
