# Supplementary material for: Effect of childhood developmental coordination disorder on adulthood physical activity; Arvo Ylppö longitudinal study
Source: Scand J Med Sci Sports. 2022 Feb 24;32(6):1050–63. doi: 10.1111/sms.14144 (PMC9306991; doi:10.1111/sms.14144)
Supplement: Supplementary file 7 — Appendix F [file SMS-32-1050-s008.docx]

### **Appendix F Differences in accelerometry measures between risk categories, with subanalysis by sex**

**DCD risk groups**

### **Table 7: Accelerometry differences between DCD risk groups**

|  | *DCD5* | *DCD15* | *Not at risk* |  | |
| --- | --- | --- | --- | --- | --- |
|  | *M (SD)* | *M (SD)* | *M (SD)* | *H statistic* | *P* |
| **Male** | *N=23* | *N=43* | *N=250* |  |  |

| Sedentary Light | 842.2 (123.6) | 873.0 (91.8) | 839.1 (109.1) | 3.3 | .193 |
| --- | --- | --- | --- | --- | --- |
| Moderate | 124.2 (64.8) | 136.0 (67.5) | 150.5 (84.4) | 2.1 | .346 |
| Vigorous | 5.7 (6.9) | 6.8 (8.4) | 7.0 (8.9) | 0.3 | .852 |
| MVPA | 129.9 (67.0) | 142.7 (70.5) | 157.5 (88.8) | 2.0 | .366 |
| % sedentary light activity | 27.6 (4.8) | 27.1 (3.6) | 27.1 (4.0) | 2.2 | .327 |
| % moderate activity | 9.1 (4.5) | 9.8 (4.8) | 11.0 (6.1) | 2.3 | .317 |
| % vigorous activity | 0.4 (0.5) | 0.5 (0.6) | 0.5 (0.6) | 0.3 | .862 |
| % MVPA | 9.6 (4.6) | 10.2 (5.0) | 11.5 (6.4) | 2.2 | .338 |
| Steps | 8776.8 (3412.0) | 9369.7 (3635.4) | 9677.5 (3729.5) | 1.4 | .493 |
| Mean amplitude deviation | 0.97 (0.3) | 0.97 (0.3) | 0.98 (0.3) | 0.2 | .918 |
| **Female** | *N=7* | *N=10* | *N=325* |  |  |
| Sedentary Light | 846.3 (120.7) | 868.6 (100.9) | 834.6 (102.4) | 1.7 | .424 |
| Moderate | 147.3 (85.7) | 107.8 (53.3) | 130.3 (73.5) | 1.7 | .433 |
| Vigorous | 4.53 (3.8) | 4.7 (4.1) | 6.3 (7.7) | 0.03 | .985 |
| MVPA | 151.8 (86.6) | 112.5 (54.1) | 136.6 (76.3) | 1.5 | .465 |
| % sedentary light activity | 63.2 (7.9) | 63.2 (4.1) | 61.0 (6.1) | 1.6 | .447 |
| % moderate activity | 10.8 (6.1) | 7.8 (3.7) | 9.5 (5.4) | 1.8 | .412 |
| % vigorous activity | 0.3 (0.3) | 0.3 (0.3) | 0.5 (0.6) | 0.1 | .974 |
| % MVPA | 11.2 (6.2) | 8.1 (3.7) | 10.0 (5.6) | 1.7 | .433 |
| Steps | 10316.7 (2202.5) | 9725.6 (2489.4) | 10842.1 (3496.2) | 0.8 | .675 |
| Mean amplitude deviation | 0.96 (0.2) | 0.93 (0.2) | 0.99 (0.2) | 0.51 | .776 |

A=T-test

### **Table 8: Accelerometry group difference between at risk of DCD (under 5^th^ percentile) and those not at risk**

|  | DCD | Not at risk | Group difference | | |
| --- | --- | --- | --- | --- | --- |
|  | *M (SD)* | *M (SD)* | ***d_Cohen_*** | U-statistic | p |
| **Males** | *N=23* | *N=250* |  |  |  |
| Age (yrs) | 24.8 (0.7) | 24.8 (0.8) | 0.001 | 0.004^†^ | .969 |
| BMI | 25.6 (4.5) | 24.5 (4.0) | 0.2 | 2388.5 | .179 |
| Sedentary light (mins) | 842.2 (123.6) | 839.1 (109.1) | 0.01 | 2847.0 | .938 |
| Moderate (mins) | 121.2 (64.8) | 150.5 (84.4) | 0.2 | 2430.0 | .219 |
| Vigorous (mins) | 5.7 (6.9) | 7.0 (8.9) | 0.1 | 2671.5 | .574 |
| MVPA (mins) | 129.9 (67.0) | 157.5 (88.8) | 0.1 | 2445.0 | .235 |
| % sedentary light activity | 62.8 (5.9) | 61.4 (6.9) | 0.1 | 2575.0 | .408 |
| % moderate activity | 9.1 (4.5) | 11.0 (6.1) | 0.1 | 2460.0 | .252 |
| % vigorous activity | 0.4 (0.5) | 0.5 (0.6) | 0.1 | 2679.0 | .589 |
| % MVPA | 9.6 (4.6) | 11.5 (6.4) | 0.1 | 2463.0 | .256 |
| Steps | 8776.8 (3411.9) | 9677.5 (3729.5) | 0.1 | 2458.0 | .250 |
| Mean amplitude deviation | 0.97 (0.3) | 0.98 (0.3) | 0.02 | 2831.0 | .903 |
| **Females** | *N=7* | *N=325* |  | | |
| Age (yrs) | 25.1 (0.9) | 24.8 (0.7) | 0.3 | 0.9^†^ | .395 |
| BMI | 26.5 (10.1) | 23.5 (4.3) | 0.02 | 1092.0 | .856 |
| Sedentary light (mins) | 846.3 (120.7) | 834.6 (102.4) | 0.01 | 1120.0 | .944 |
| Moderate (mins) | 147.3 (85.7) | 130.3 (73.5) | 0.1 | 970.0 | .505 |
| Vigorous (mins) | 4.5 (3.8) | 6.3 (7.7) | 0.0 | 1136.5 | .997 |
| MVPA (mins) | 151.8 (86.6) | 136.6 (76.3) | 0.1 | 982.0 | .536 |
| % sedentary light activity | 63.2 (7.9) | 61.0 (6.1) | 0.1 | 1000.0 | .584 |
| % moderate activity | 10.8 (6.1) | 9.5 (5.4) | 0.1 | 971.0 | .508 |
| % vigorous activity | 0.3 (0.3) | 0.5 (0.6) | 0.01 | 1120.0 | .944 |
| % MVPA | 11.2 (6.2) | 10.0 (5.6) | 0.1 | 994.0 | .568 |
| Steps | 10316.7 (2202.5) | 10842.1 (3496.2) | 0.02 | 1101.0 | .884 |
| Mean amplitude deviation | 0.96 (0.2) | 1.0 (0.2) | 0.05 | 1030.0 | .669 |

†=t-test

### **Table 9: Accelerometry group difference between probably at risk of DCD (5^th^ to 15^th^ percentile) and those not at risk**

|  | DCD | Not at risk | Group difference | | |
| --- | --- | --- | --- | --- | --- |
|  | *M (SD)* | *M (SD)* | ***d_Cohen_*** | U-statistic | P |
| **Males** | *N=43* | *N=250* |  |  |  |
| Age (yrs) | 24.9 (0.6) | 24.8 (0.8) | 0.1 | 0.73^†^ | .467 |
| BMI | 24.7 (4.4) | 24.5 (4.0) | 0.001 | 5372.0 | .995 |
| Sedentary light (mins) | 873.0 (91.8) | 839.1 (109.1) | 0.2 | 4444.0 | .070 |
| Moderate (mins) | 136.0 (67.5) | 150.5 (84.4) | 0.1 | 4904.5 | .359 |
| Vigorous (mins) | 6.8 (8.4) | 7.0 (8.9) | 0.01 | 5342.0 | .949 |
| MVPA (mins) | 142.7 (70.5) | 157.5 (88.8) | 0.1 | 4914.5 | .370 |
| % sedentary light activity | 62.7 (6.3) | 61.4 (6.9) | 0.2 | 4692.0 | .183 |
| % moderate activity | 9.8 (4.8) | 11.0 (6.1) | 0.1 | 4803.00 | .265 |
| % vigorous activity | 0.5 (0.6) | 0.2 (0.6) | 0.01 | 5351.50 | .963 |
| % MVPA | 10.2 (5.0) | 11.5 (6.4) | 0.1 | 4835.0 | .293 |
| Steps | 9369.7 (3635.4) | 9677.5 (3729.5) | 0.1 | 5134.0 | .639 |
| Mean amplitude deviation | 0.97 (0.3) | 0.98 (0.3) | 0.1 | 5162.0 | .678 |
| **Females** | N=10 | N=325 |  | | |
| Age (yrs) | 24.9 (0.6) | 24.8 (0.7) | 0.1 | 0.4^†^ | .066 |
| BMI | 24.7 (5.0) | 23.5 (4.3) | 0.1 | 1274.5 | .245 |
| Sedentary light (mins) | 868.6 (100.9) | 834.6 (102.4) | 0.1 | 1230.0 | .190 |
| Moderate (mins) | 107.8 (53.3) | 130.3 (73.5) | 0.1 | 1295.0 | .273 |
| Vigorous (mins) | 4.7 (4.1) | 6.3 (7.7) | 0.02 | 1573.0 | .863 |
| MVPA (mins) | 112.5 (54.1) | 136.6 (76.3) | 0.1 | 1304.0 | .287 |
| % sedentary light activity | 63.2 (4.1) | 61.0 (6.1) | 0.1 | 1275.0 | .246 |
| % moderate activity | 7.8 (3.7) | 0.5 (5.4) | 0.1 | 1279.0 | .251 |
| % vigorous activity | 0.3 (0.3) | 0.5 (0.6) | 0.02 | 1559.0 | .827 |
| % MVPA | 8.1 (3.7) | 10.0 (5.6) | 0.1 | 1278.0 | .250 |
| Steps | 9725.6 (2489.4) | 10842.1 (33496.2) | 0.1 | 1362.0 | .383 |
| Mean amplitude deviation | 0.93 (0.2) | 1.00 (0.2) | 0.1 | 1450.0 | .562 |

A=T-test

### **Table 10: Accelerometry group difference between DCD risk group (DCD5 and 15) and not at risk**

|  | DCD | Not at risk | Group difference | | |
| --- | --- | --- | --- | --- | --- |
|  | *M (SD)* | *M (SD)* | ***d_Cohen_*** | U-statistic | p |
| **Males** | *N=66* | *N=250* |  |  |  |
| Age (yrs) | 24.9 (0.6) | 24.8 (0.8) | 0.1 | 0.7^†^ | .514 |
| BMI | 25.0 (4.4) | 24.5 (4.0) | 0.1 | 7766.5 | .464 |
| Sedentary light (mins) | 862.3 (104.1) | 839.1 (109.1) | 0.2 | 7291.0 | .146 |
| Moderate (mins) | 131.9 (66.3) | 150.5 (84.4) | 0.2 | 7334.5 | .166 |
| Vigorous (mins) | 6.4 (7.9) | 7.0 (8.9) | 0.03 | 8079.5 | .796 |
| MVPA (mins) | 138.3 (69.1) | 157.5 (88.8) | 0.2 | 7359.5 | .177 |
| % sedentary light activity | 62.7 (6.1) | 61.4 (6.9) | 0.2 | 7267.0 | .137 |
| % moderate activity | 9.5 (4.7) | 11.0 (6.1) | 0.2 | 7263.0 | .135 |
| % vigorous activity | 0.5 (0.6) | 0.5 (0.6) | 0.04 | 8030.5 | .740 |
| % MVPA | 10.0 (4.9) | 11.5 (6.4) | 0.2 | 7298.0 | .149 |
| Steps | 9163.1 (3544.2) | 9677.5 (3729.5) | 0.1 | 7592.0 | .319 |
| Mean amplitude deviation | 0.97 (0.3) | 0.98 (0.3) | 0.04 | 7993.0 | .697 |
| **Females** | *N=17* | *N=325* |  |  |  |
| Age (yrs) | 25.0 (0.7) | 24.8 (0.7) | 0.2 | 0.9^†^ | .360 |
| BMI | 25.4 (7.3) | 23.5 (4.3) | 0.1 | 2457.5 | .443 |
| Sedentary light (mins) | 859.4 (106.4) | 834.6 (102.4) | 0.1 | 2385.0 | .342 |
| Moderate (mins) | 124.1 (68.9) | 130.3 (73.5) | 0.04 | 2600.0 | .683 |
| Vigorous (mins) | 4.6 (3.9) | 6.3 (7.7) | 0.01 | 2709.5 | .894 |
| MVPA (mins) | 128.7 (69.7) | 136.6 (76.3) | 0.05 | 2597.0 | .677 |
| % sedentary light activity | 63.2 (5.7) | 61.0 (6.1) | 0.13 | 2275.0 | .220 |
| % moderate activity | 9.1 (4.9) | 9.5 (5.4) | 0.05 | 2583.0 | .651 |
| % vigorous activity | 0.3 (0.3) | 0.5 (0.6) | 0.01 | 2714.0 | .903 |
| % MVPA | 9.4 (4.9) | 10.0 (5.6) | 0.1 | 2559.0 | .609 |
| Steps | 9969.0 (2322.7) | 10842.1 (3496.2) | 0.1 | 2463.0 | .451 |
| Mean amplitude deviation | 0.9 (0.2) | 1.0 (0.2) | 0.1 | 2480.0 | .477 |

†=T-test

**VMI risk categories**

### **Table 11: Accelerometry group difference between Berry under 5^th^ percentile and those above the 15^th^ percentile**

|  | <5^th^ percentile | >15^th^ percentile | Group difference | | |
| --- | --- | --- | --- | --- | --- |
|  | *M (SD)* | *M (SD)* | ***d_Cohen_*** | U-statistic | p |
| **Males** | *N=16* | *N=272* |  |  |  |
| Age (yrs) | 25.3 (0.8) | 24.8 (0.7) | 0.7 | 2.6^†^ | .021 |
| BMI | 26.5 (4.8) | 24.5 (3.9) | 0.3 | 1491.0 | .034 |
| Sedentary light (mins) | 891.3 (80.2) | 838.9 (109.9) | 0.2 | 1588.5 | .070 |
| Moderate (mins) | 124.1 (64.9) | 148.4 (83.0) | 0.1 | 1793.5 | .237 |
| Vigorous (mins) | 3.1 (7.8) | 7.3 (9.0) | 0.3 | 1237.5 | .004 |
| MVPA (mins) | 127.2 (71.1) | 155.7 (87.2) | 0.2 | 1721.5 | .160 |
| % sedentary light activity | 64.7 (4.4) | 61.3 (6.8) | 0.2 | 1519.0 | .042 |
| % moderate activity | 8.9 (4.5) | 10.8 (6.0) | 0.2 | 1764.0 | .203 |
| % vigorous activity | 0.2 (0.6) | 0.5 (0.7) | 0.3 | 1223.5 | .003 |
| % MVPA | 9.1 (5.0) | 11.4 (6.3) | 0.2 | 1691.0 | .134 |
| Steps | 9355.1 (3727.3) | 9638.7 (3730.2) | 0.04 | 2065.0 | .732 |
| Mean amplitude deviation | 0.94 (0.2) | 0.98 (0.3) | 0.1 | 2003.0 | .593 |
| **Females** | *N=7* | *N=309* |  | | |
| Age (yrs) | 25.1 (0.9) | 24.8 (0.7) | 0.3 | 0.9^†^ | .412 |
| BMI | 24.9 (5.9) | 23.7 (4.5) | 0.05 | 973.5 | .651 |
| Sedentary light (mins) | 831.4 (108.9) | 837.1 (104.2) | 0.04 | 992.5 | .710 |
| Moderate (mins) | 122.3 (55.8) | 128.5 (74.1) | 0.02 | 1050.0 | .895 |
| Vigorous (mins) | 7.9 (10.2) | 6.0 (7.2) | 0.03 | 1028.5 | .824 |
| MVPA (mins) | 130.1 (62.8) | 134.5 (76.5) | 0.02 | 1040.5 | .864 |
| % sedentary light activity | 59.9 (6.9) | 61.3 (6.1) | 0.07 | 943.0 | .562 |
| % moderate activity | 8.9 (4.1) | 9.4 (5.4) | 0.01 | 1057.0 | .918 |
| % vigorous activity | 0.56 (0.73) | 0.4 (0.5) | 0.02 | 1030.5 | .813 |
| % MVPA | 9.4 (4.6) | 9.9 (5.6) | 0.01 | 1053.0 | .905 |
| Steps | 10955.4 (1879.5) | 10710.6 (3477.2) | 0.1 | 956.0 | .600 |
| Mean amplitude deviation | 1.1 (0.2) | 1.0 (0.2) | 0.2 | 728.0 | .139 |

†=T-test

### **Table 12: Accelerometry group difference between Berry 5^th^ to 15^th^ percentile and those above the 15^th^ percentile**

|  | 5-15^th^ percentile | >15^th^ percentile | Group difference | | |
| --- | --- | --- | --- | --- | --- |
|  | *M (SD)* | *M (SD)* | ***d_Cohen_*** | U-statistic | P |
| **Males** | *N=18* | *N=272* |  |  |  |
| Age (yrs) | 24.9 (0.6) | 24.8 (0.7) | 0.1 | 0.5^†^ | .588 |
| BMI | 24.8 (5.5) | 24.5 (3.9) | 0.02 | 2379.0 | .841 |
| Sedentary light (mins) | 850.1 (119.0) | 838.9 (109.9) | 0.02 | 2399.5 | .888 |
| Moderate (mins) | 159.7 (92.5) | 148.4 (90.0) | 0.05 | 2304.0 | .676 |
| Vigorous (mins) | 7.3 (8.1) | 7.3 (9.0) | 0.01 | 2430.5 | .959 |
| MVPA (mins) | 167.1 (95.5) | 155.7 (87.2) | 0.06 | 2283.0 | .632 |
| % sedentary light activity | 61.7 (8.3) | 61.3 (6.8) | 0.03 | 2352.0 | .781 |
| % moderate activity | 11.6 (6.7) | 10.8 (6.0) | 0.04 | 2328.0 | .728 |
| % vigorous activity | 0.5 (0.6) | 0.5 (0.7) | 0.002 | 2443.0 | .988 |
| % MVPA | 12.1 (6.9) | 11.4 (6.3) | 0.05 | 2300.0 | .668 |
| Steps | 9522.8 (4106.1) | 9638.7 (3730.2) | 0.05 | 2309.0 | .687 |
| Mean amplitude deviation | 1.0 (0.3) | 0.98 (0.3) | 0.04 | 2329.0 | .730 |
| **Female** | *N=14* | *N=309* |  | | |
| Age (yrs) | 24.7 (0.5) | 24.8 (0.7) | 0.3 | 1.0^†^ | .350 |
| BMI | 21.3 (2.2) | 23.7 (4.5) | 0.2 | 1466.0 | .041 |
| Sedentary light (mins) | 808.3 (71.0) | 837.1 (104.2) | 0.2 | 1662.5 | .114 |
| Moderate (mins) | 169.2 (63.8) | 128.5 (74.1) | 0.3 | 1261.0 | .008 |
| Vigorous (mins) | 9.1 (11.5) | 6.0 (7.2) | 0.1 | 1843.0 | .349 |
| MVPA (mins) | 178.2 (72.1) | 134.5 (76.5) | 0.3 | 1299.5 | .012 |
| % sedentary light activity | 59.0 (4.8) | 61.3 (6.1) | 0.2 | 1573.0 | .084 |
| % moderate activity | 12.3 (4.5) | 9.4 (5.4) | 0.3 | 1268.0 | .009 |
| % vigorous activity | 0.7 (0.8) | 0.4 (0.5) | 0.1 | 1854.5 | .367 |
| % MVPA | 13.0 (5.1) | 9.9 (5.6) | 0.3 | 1314.0 | .013 |
| Steps | 12574.8 (3560.6) | 10710.6 (3477.2) | 0.2 | 1478.0 | .045 |
| Mean amplitude deviation | 1.11 (0.3) | 0.99 (0.2) | 0.2 | 1670.0 | .149 |

†=T-test

### **Table 13: Accelerometry group difference between Berry under 15^th^ percentile and those above the 15^th^ percentile**

|  | <15^th^ percentile | >15^th^ percentile | Group difference | | |
| --- | --- | --- | --- | --- | --- |
|  | *M (SD)* | *M (SD)* | ***d_Cohen_*** | U-statistic | P |
| **Males** | *N=34* | *N=282* |  |  |  |
| Age (yrs) | *25.1 (0.7)* | *24.8 (0.7)* | ***0.02*** | 2.3^†^ | .029 |
| BMI | 25.6 (5.2) | 24.5 (3.9) | 0.2 | 3870.0 | .121 |
| Sedentary light (mins) | 869.5 (103.2) | 838.9 (109.9) | 0.2 | 3988.0 | .191 |
| Moderate (mins) | 143.0 (81.5) | 148.4 (83.0) | 0.1 | 4385.5 | .624 |
| Vigorous (mins) | 5.3 (8.1) | 6.3 (9.0) | 0.2 | 3703.0 | .058 |
| MVPA (mins) | 148.3 (86.1) | 155.7 (87.2) | 0.1 | 4334.5 | .552 |
| % sedentary light activity | 63.1 (6.8) | 61.3 (6.8) | 0.2 | 3871.0 | .122 |
| % moderate activity | 10.3 (5.8) | 10.8 (6.0) | 0.1 | 4332.0 | .548 |
| % vigorous activity | 0.4 (0.6) | 0.5 (0.7) | 1.2 | 366.5 | .049 |
| % MVPA | 10.7 (6.2) | 11.4 (6.3) | 0.1 | 4287.0 | .488 |
| Steps | 9443.9 (3874.0) | 9638.7 (3730.2) | 0.1 | 4374.0 | .607 |
| Mean amplitude deviation | 0.98 (0.25) | 0.98 (0.28) | 0.01 | 4570.0 | .912 |
| **Females** | *N=21* | *N=309* |  | | |
| Age (yrs) | 24.9 (0.7) | 24.8 (0.7) | 0.02 | 0.1^†^ | .917 |
| BMI | 22.4 (4.1) | 23.7 (4.5) | 0.2 | 2655.5 | .164 |
| Sedentary light (mins) | 816.0 (83.4) | 837.1 (104.2) | 0.2 | 2615.0 | .137 |
| Moderate (mins) | 153.5 (64.0) | 128.5 (74.1) | 0.2 | 2311.0 | .027 |
| Vigorous (mins) | 8.7 (10.8) | 6.0 (7.2) | 0.1 | 2871.5 | .378 |
| MVPA (mins) | 162.2 (71.4) | 134.5 (76.5) | 0.2 | 2340.0 | .033 |
| % sedentary light activity | 59.3 (5.4) | 61.3 (6.1) | 0.2 | 2516.0 | .085 |
| % moderate activity | 11.2 (4.6) | 9.4 (5.4) | 0.2 | 2325.0 | .030 |
| % vigorous activity | 0.6 (0.8) | 0.4 (0.5) | 0.1 | 2885.0 | .395 |
| % MVPA | 11.8 (5.1) | 9.9 (5.6) | 0.2 | 2367.0 | .038 |
| Steps | 12035.0 (3148.4) | 10710.6 (3477.2) | 0.2 | 2434.0 | .055 |
| Mean amplitude deviation | 1.11 (0.3) | 0.99 (0.2) | 0.2 | 2398.0 | .045 |

†=T-test
