# Supplementary material for: Effect of childhood developmental coordination disorder on adulthood physical activity; Arvo Ylppö longitudinal study
Source: Scand J Med Sci Sports. 2022 Feb 24;32(6):1050–63. doi: 10.1111/sms.14144 (PMC9306991; doi:10.1111/sms.14144)
Supplement: Supplementary file 8 — Appendix G [file SMS-32-1050-s003.docx]

## Appendix G

Sensitivity analysis based on four days wear

### **Table 14: Accelerometry group difference between DCD (DCD5 and 15) and not at risk**

|  | DCD  N=80 | Not at risk  N=570 | Group difference | | |
| --- | --- | --- | --- | --- | --- |
|  | *M (SD) [Md]* | *M (SD)* | ***d_Cohen_*** | U-statistic | p |
| Age (yrs) | 24.9 (0.6) [25.0] | 24.8 (0.7) [25.0] | -0.1 | 21465.5 | .359 |
| BMI | 24.9 (5.0) [23.8] | 23.9 (4.2) [23.1] | -0.2 | 19890.5 | .064 |
| Sedentary light (mins/day) | 860.9 (104.5) [866.2] | 837.8 (103.1) [853.9] | -0.2 | 20087.0 | .085 |
| Moderate (mins/day) | 130.6 (67.4) [118.8] | 139.5 (79.1) [122.4] | 0.1 | 21750.0 | .504 |
| Vigorous (mins/day) | 5.8 (6.9) [3.6] | 6.6 (8.3) [3.6] | 0.1 | 22065.5 | .640 |
| MVPA (mins/day) | 136.3 (69.8) [124.1] | 146.1 (82.6) [129.2] | 0.1 | 21635.5 | .459 |
| % Sedentary light activity | 62.8 (6.0) [63.8] | 61.2 (6.4) [61.8] | -0.3 | 19352.0 | .028 |
| % Moderate activity | 9.5 (4.7) [8.6] | 10.2 (5.8) [8.9] | 0.1 | 21642.0 | .462 |
| % Vigorous activity | 0.4 (0.5) [0.3] | 0.5 (0.6) [0.3] | 0.2 | 22008.0 | .615 |
| % MVPA | 9.9 (4.9) [8.9] | 10.7 (6.0) [9.4] | 0.1 | 21516.0 | .414 |
| Steps | 9213.7 (3247.9) [9035.0] | 10362.7 (3637.7) [9958.0] | 0.3 | 18774.0 | .010 |
| Mean amplitude deviation | 0.96 (0.3) [0.92] | 0.97 (0.3)[0.96] | 0.03 | 21064.0 | .270 |

## **Table 15: Accelerometry complex models for physical activity with DCD as a probable risk factor**

|  |  |  |  | β 95% Confidence interval | |  |
| --- | --- | --- | --- | --- | --- | --- |
| Model |  | β | S.E. | Lower | Upper | P |
| Sedentary light | Intercept | 6.5 | 0.03 | 6.4 | 6.6 | <.001 |
|  | Sex^†^ | -0.01 | 0.01 | -0.03 | 0.01 | .328 |
|  | Mother’s education (secondary)^§^ | -0.01 | 0.02 | -0.04 | 0.02 | .518 |
|  | Mother’s education (upper secondary) ^¶^ | -0.01 | 0.02 | -0.04 | 0.02 | .477 |
|  | Mother’s education (Masters)^††^ | -0.01 | 0.01 | -0.03 | 0.02 | .637 |
|  | DCD^‡^ | 0.2 | 0.1 | 0.02 | 0.3 | .022 |
|  | BMI | 0.01 | 0.001 | 0.007 | 0.01 | <.001 |
|  | BMI*DCD interaction effect | -0.01 | 0.003 | -0.01 | 0.0 | .048 |
| Moderate | Intercept | 6.4 | 0.1 | 6.2 | 6.7 | <.001 |
|  | Sex^†^ | 0.2 | 0.04 | 0.1 | 0.3 | <.001 |
|  | Mother’s education (secondary)^§^ | 0.01 | 0.1 | -0.1 | 0.1 | .855 |
|  | Mother’s education (upper secondary) ^¶^ | 0.04 | 0.1 | -0.1 | 0.2 | .515 |
|  | Mother’s education (Masters)^††^ | 0.05 | 0.05 | -0.1 | 0.1 | .351 |
|  | DCD^‡^ | -0.5 | 0.3 | -1.1 | 0.1 | .130 |
|  | BMI | -0.1 | 0.01 | -0.08 | -0.06 | <.001 |
|  | BMI* DCD interaction effect | 0.02 | 0.01 | -0.01 | 0.04 | .187 |
| Vigorous | Intercept | 3.5 | 0.2 | 3.1 | 3.9 | <.001 |
|  | Sex^†^ | 0.1 | 0.1 | -0.04 | 0.3 | .143 |
|  | Mother’s education (secondary)^§^ | -0.2 | 0.1 | -0.4 | 0.1 | .184 |
|  | Mother’s education (upper secondary) ^¶^ | -0.1 | 0.1 | -0.3 | 0.2 | .529 |
|  | Mother’s education (Masters)^††^ | 0.01 | 0.1 | -0.2 | 0.2 | .892 |
|  | DCD^‡^ | -0.9 | 0.5 | -1.9 | 0.05 | .062 |
|  | BMI | -0.1 | 0.01 | -0.1 | -0.06 | <.001 |
|  | BMI* DCD interaction effect | 0.04 | 0.02 | -0.002 | 0.07 | .063 |
| MVPA | Intercept | 6.5 | 0.1 | 6.3 | 6.8 | <.001 |
|  | Sex^†^ | 0.2 | 0.04 | 0.1 | 0.3 | <.001 |
|  | Mother’s education (secondary)^§^ | 0.0 | 0.1 | -0.1 | 0.1 | .965 |
|  | Mother’s education (upper secondary) ^¶^ | 0.03 | 0.1 | -0.1 | 0.1 | .643 |
|  | Mother’s education (Masters)^††^ | 0.04 | 0.1 | -0.1 | 0.1 | .393 |
|  | DCD^‡^ | -0.5 | 0.3 | -1.1 | 0.1 | .098 |
|  | BMI | -0.1 | 0.01 | -0.1 | -0.07 | <.001 |
|  | BMI* DCD | 0.02 | 0.01 | -0.01 | 0.04 | .147 |
| Steps | Intercept | 9.6 | 0.1 | 9.4 | 9.8 | <.001 |
|  | Sex^†^ | -0.1 | 0.03 | -0.2 | -0.1 | <.001 |
|  | Mother’s education (secondary)^§^ | -0.01 | 0.1 | -0.1 | 0.01 | .784 |
|  | Mother’s education (upper secondary) ^¶^ | -0.04 | 0.04 | -0.1 | 0.04 | .347 |
|  | Mother’s education (Masters)^††^ | 0.03 | 0.04 | -0.05 | 0.1 | .470 |
|  | DCD^‡^ | -0.2 | 0.02 | -0.6 | 0.2 | .261 |
|  | BMI | -0.01 | 0.004 | -0.02 | -0.01 | <.001 |
|  | BMI* DCD interaction effect | 0.01 | 0.01 | -0.01 | 0.02 | .419 |
| Mean amplitude deviation | Intercept | 0.4 | 0.1 | 0.2 | 0.5 | <.001 |
|  | Sex^†^ | -0.01 | 0.02 | -0.1 | 0.04 | .827 |
|  | Mother’s education (secondary)^§^ | 0.01 | 0.03 | -0.1 | 0.1 | .660 |
|  | Mother’s education (upper secondary) ^¶^ | 0.01 | 0.03 | -0.1 | 0.1 | .787 |
|  | Mother’s education (Masters)^††^ | 0.01 | 0.03 | -0.04 | 0.1 | .601 |
|  | DCD^‡^ | -0.02 | 0.2 | -0.3 | 0.3 | .876 |
|  | BMI | -0.02 | 0.003 | -0.02 | -0.01 | <.001 |
|  | BMI* DCD interaction effect | 0.0 | 0.01 | -0.01 | 0.01 | .972 |

† Where male is the comparison group and β=1; ‡ Where DCD is the comparison group and β=1; § Where education is level 1; ¶ Where education is level 2; †† Where education is level 3

### **Table 16: Accelerometry differences based on VMI risk status (15^th^ percentile)**

|  | Less than 15%  N=54 | Greater than 15%  N=574 | Group difference | | |
| --- | --- | --- | --- | --- | --- |
|  | *M (SD) [Md]* | *M (SD) [Md]* | ***d_Cohen_*** | U statistic | p |
| Age (yrs) | 25.0 (0.7) [25.0] | 24.8 (0.7) [25.0] | -0.3 | 13304.5 | .063 |
| BMI | 24.1 (4.7) [23.4] | 24.0 (4.3) [23.2] | -0.02 | 15247.5 | .844 |
| Sedentary light (min/day) | 846.8 (98.4) [828.6] | 839.2 (104.8) [856.9] | -0.1 | 13504.0 | .980 |
| Moderate (min/day) | 148.2 (75.1) [134.4] | 138.2 (79.2) [121.8] | -0.1 | 13955.0 | .226 |
| Vigorous (min/day) | 6.7 (9.3) [2.8] | 6.6 (8.1) [3.8] | -0.01 | 14411.0 | .394 |
| Moderate and vigorous (min/day) | 154.9 (80.6) [138.9] | 144.8 (82.6) [128.0] | -0.1 | 14153.5 | .292 |
| % sedentary light activity | 61.5 (6.5) [62.4] | 61.3 (6.5) [62.0] | -0.03 | 15285.0 | .867 |
| % moderate activity | 10.7 (5.4) [9.6] | 10.1 (5.7) [8.8] | -0.1 | 14068.0 | .262 |
| % vigorous activity | 0.5 (0.7) [0.2] | 0.5 (0.6) [0.3] | 0.0 | 14350.0 | .368 |
| % moderate and vigorous activity | 11.2 (5.8) [9.9] | 10.6 (6.0) [9.3] | -0.1 | 14239.0 | .323 |
| Steps | 10492.1 (3812.5) [9931.9] | 10217.9 (3623.8) [9488.9] | -0.1 | 14939.0 | .063 |
| Mean amplitude deviation | 1.03 (0.3) [1.0] | 1.0 (0.3) [1.0] | -0.1 | 14196.0 | .307 |

†=t-test

### **Table 17: Accelerometry complex models for physical activity for VMI categorised on the 15th percentile**

|  |  |  |  | 95% Confidence interval | |  |
| --- | --- | --- | --- | --- | --- | --- |
| Model |  | β | S.E. | Lower | Upper | P |
| Sedentary light | Intercept | 6.5 | 0.03 | 6.5 | 6.6 | <.001 |
|  | Sex^†^ | -0.01 | 0.01 | -0.03 | 0.01 | .411 |
|  | Mother’s education (secondary)^§^ | -0.01 | 0.02 | -0.1 | 0.02 | .443 |
|  | Mother’s education (upper secondary)^¶^ | -0.01 | 0.02 | -0.04 | 0.02 | .543 |
|  | Mother’s education (Masters)^††^ | 0.0 | 0.01 | -0.03 | 0.02 | .813 |
|  | VMI category^‡^ | -0.01 | 0.1 | -0.2 | 0.1 | .897 |
|  | BMI | 0.01 | 0.001 | 0.006 | 0.01 | <.001 |
|  | BMI*VMI interaction | 0.001 | 0.003 | -0.01 | 0.01 | .764 |
| Moderate | Intercept | 6.4 | 0.1 | 6.1 | 6.6 | <.001 |
|  | Sex^†^ | 0.2 | 0.04 | 0.1 | 0.2 | <.001 |
|  | Mother’s education (secondary)^§^ | 0.03 | 0.1 | -0.1 | 0.1 | .690 |
|  | Mother’s education (upper secondary)^¶^ | 0.02 | 0.1 | -0.1 | 0.1 | .695 |
|  | Mother’s education (Masters)^††^ | 0.03 | 0.05 | -0.1 | 0.1 | .558 |
|  | VMI category^‡^ | -0.2 | 0.3 | -0.8 | 0.4 | .419 |
|  | BMI | -0.1 | 0.01 | -0.1 | -0.06 | <.001 |
|  | BMI*VMI interaction | 0.01 | 0.01 | -0.01 | 0.04 | .320 |
| Vigorous | Intercept | 3.4 | 0.2 | 3.0 | 3.8 | <.001 |
|  | Sex^†^ | 0.1 | 0.1 | -0.03 | 0.3 | .112 |
|  | Mother’s education (secondary)^§^ | -0.2 | 0.1 | -0.5 | 0.04 | .105 |
|  | Mother’s education (upper secondary)^¶^ | -0.1 | 0.1 | -0.3 | 0.1 | .444 |
|  | Mother’s education (Masters)^††^ | 0.0 | 0.1 | -0.2 | 0.2 | 1.000 |
|  | VMI category^‡^ | 0.3 | 0.6 | -0.8 | 1.4 | .622 |
|  | BMI | -0.1 | 0.01 | -0.1 | -0.06 | <.001 |
|  | BMI*VMI interaction | -0.02 | 0.02 | -0.1 | 0.03 | .483 |
| MVPA | Intercept | 6.5 | 0.1 | 6.2 | 6.7 | <.001 |
|  | Sex^†^ | 0.2 | 0.04 | 0.1 | 0.2 | <.001 |
|  | Mother’s education (secondary)^§^ | 0.01 | 0.1 | -0.1 | 0.1 | .838 |
|  | Mother’s education (upper secondary)^¶^ | 0.01 | 0.1 | -0.1 | 0.1 | .843 |
|  | Mother’s education (Masters)^††^ | 0.03 | 0.1 | -0.08 | 0.1 | .618 |
|  | VMI category^‡^ | -0.2 | 0.3 | -0.8 | 0.4 | .465 |
|  | BMI | -0.1 | 0.01 | -0.1 | -0.06 | <.001 |
|  | BMI*VMI interaction | 0.01 | 0.01 | -0.01 | 0.04 | .371 |
| Steps | Intercept | 9.6 | 0.1 | 9.4 | 9.8 | <.001 |
|  | Sex^†^ | -0.1 | 0.03 | -0.2 | -0.1 | <.001 |
|  | Mother’s education (secondary)^§^ | -0.01 | 0.05 | -0.1 | 0.01 | .798 |
|  | Mother’s education (upper secondary)^¶^ | -0.05 | 0.04 | -0.1 | 0.04 | .267 |
|  | Mother’s education (Masters)^††^ | 0.02 | 0.04 | -0.1 | 0.1 | .666 |
|  | VMI category^‡^ | -0.3 | 0.2 | -0.8 | 0.2 | .200 |
|  | BMI | -0.02 | 0.004 | -0.02 | -0.01 | <.001 |
|  | BMI*VMI interaction | 0.02 | 0.01 | -0.01 | 0.04 | .137 |
| Mean amplitude deviation | Intercept | 0.4 | 0.1 | 0.3 | 0.5 | <.001 |
|  | Sex^†^ | -0.02 | 0.02 | -0.1 | 0.03 | .478 |
|  | Mother’s education (secondary)^§^ | 0.01 | 0.03 | -0.1 | 0.1 | .704 |
|  | Mother’s education (upper secondary)^¶^ | -0.002 | 0.03 | -0.1 | 0.1 | .957 |
|  | Mother’s education (Masters)^††^ | 0.01 | 0.03 | -0.04 | 0.1 | .697 |
|  | VMI category^‡^ | -0.3 | 0.2 | -0.6 | 0.1 | .099 |
|  | BMI | -0.02 | 0.003 | -0.02 | -0.01 | <.001 |
|  | BMI*VMI interaction | 0.01 | 0.01 | 0.001 | 0.03 | .039 |

† Where male is the comparison group and β=1; ‡ Where VMI under 15^th^ percentile is the comparison group and β=1; § Where education is level 1; ¶ Where education is level 2; †† Where education is level 3
